# Risk Characteristics of Hydrogen Sulphide Exposure in Wastewater Collection and Treatment Related Occupations

**DOI:** 10.1093/annweh/wxac065

**Published:** 2022-09-17

**Authors:** Åse Dalseth Austigard, Hans Thore Smedbold, Kristin von Hirsch Svendsen

**Affiliations:** Department of Industrial Economics and Technology Management, NTNU, Norwegian University of Science and Technology, PO Box 8900, Torgarden, N-7491 Trondheim, Norway; Trondheim Municipality, Working Environment Office, PO. Box 2300 Torgarden, N-7004 Trondheim, Norway; Department of Occupational Medicine, St Olav University Hospital, PO Box 3250, Torgarden, N-7006 Trondheim, Norway; Department of Public Health and Nursing, Faculty of Medicine and Health Sciences, NTNU, Norwegian University of Science and Technology, N-7491 Trondheim, Norway; Department of Industrial Economics and Technology Management, NTNU, Norwegian University of Science and Technology, PO Box 8900, Torgarden, N-7491 Trondheim, Norway

**Keywords:** algorithm, CV, H_2_S, hydrogen sulphide, peak, personal alarm equipment, time weighted average, wastewater

## Abstract

**Objectives:**

Water and wastewater workers can be exposed to hydrogen sulphide (H_2_S), with an unpredictable exposure pattern, dominated by sharp peaks. These peaks can often be high above the ceiling value (CV) at 10 ppm.

**Methods:**

We have analyzed self-administrated H_2_S exposure data among 60 wastewater workers in the Municipality of Trondheim, Norway, from 2015 till 2021. The detection range of the personal alarm equipment used was 1.6 to 100 ppm H_2_S. The workers were divided in four similar exposed groups (SEGs): wastewater collection net, wastewater treatment plants, wastewater pumping stations and water distribution net.

**Results:**

We identified measurements from 7083 different workdays, approximately 10% of the workdays between 2015 and 2021. Within these, 1295 days had readings above 1.6 ppm H_2_S, and 424 (33%) of these days had readings that exceeded the CV of 10 ppm H_2_S. This percentage was similar across the SEGs. Only one workday had a time weighted average (TWA) exceeding the occupational exposure limit (OEL) of 5 ppm H_2_S, and only 14 days exceeded 0.5 ppm H_2_S, 1/10th of the OEL.

**Conclusions:**

Wastewater workers in this study are regularly exposed to short peaks of H_2_S, but even high peaks do not influence the 8-h TWA values significantly. A preliminary measurement program over 3 days according to EN 689 to evaluate the need for further measurements would probably not find TWA values greater than 1/10 of OEL; the EN 689 standard is not made for evaluation to peak exposures. Exceedances of CV at 10 ppm H_2_S occur in 6% of workdays, and in 33% of days with exposure above 1.6 ppm. The toxicity and exposure profile of H_2_S makes continuous exposure monitoring necessary for alarm purposes. Reliance on the 8-h occupational exposure limit as has been the normal in Norway, will not be adequately protective for wastewater workers. H_2_S alarm equipment should continue to be used.

**What’s Important About This Paper?**

Hydrogen sulphide (H_2_S) has an acute effect on health, and alarm equipment is used to detect peak exposures that may be of immediate danger to life and health. This study used data from such alarm equipment to characterize H_2_S exposures among wastewater workers in one municipality, and demonstrated that peak exposures occur, though time weighted average exposures are well below occupational exposure limits. Importantly, the temporal patterns of the peaks mean that ongoing real-time monitoring is needed as standard sampling campaigns would be likely to miss these high-risk exposures.

## Introduction

It is well known that short-duration exposures to high concentrations of hydrogen sulphide (H_2_S)—e.g. peaks, can cause acute health effects that range in severity from eye irritation to death (Svendsen, 2001; Arbeidstilsynet, 2011; Guidotti, 2015). These peak exposures are a problem in both risk evaluation and management because they are unpredictable. As a result, personal alarm equipment has become common in wastewater work in Norway and other industries where H_2_S exposures are anticipated. The Trondheim municipality has used such equipment since 2013.

In Norway and in the European Union (EU), the 8-h occupational exposure limit value (OEL) for H_2_S exposure is 5 ppm, based on nasal effects. In addition, the EU has a short-term (15 min) exposure limit (STEL) of 10 ppm, while Norway has a ceiling value (CV) of 10 ppm, based on protection against eye irritations (Arbeidstilsynet, 2011, p 19–20). In Norway, the CV was chosen instead of a STEL, as H_2_S levels that pose immediate danger to life and health (IDLH) [e.g. 100 ppm (NIOSH, 2019)] can occur even when the 15 min average exposure is kept below 10 ppm. The time weighted average (TWA) for 8-h work exposure to H_2_S in Norway is reported to be low, mainly below 0.25 ppm (Arbeidstilsynet, 2011, p 20–21). Peak values are not reported. The urban and rural background exposure to H_2_S typically range between 0.11 parts per billion (ppb) and 0.33 ppb, although it can be as high as 1 ppb (ATSDR, 2016).

Tasks performed by water and wastewater workers that are anticipated to result in H_2_S exposures differ among the worker groups, in particular those tasks that include flushing and/or disturbances of wastewater sediments. Such disturbances are expected to be significant sources of exposure to H_2_S, as well as endotoxins (Heldal et al., 2010, 2019). In Trondheim, all flushing is done with clean water. All workers have long periods with no exposure to H_2_S, which lowers the TWA exposure levels. Outside plants, these low mean levels are mainly due to driving between exposed tasks: to refill flushing water, to empty sewage from vacuum trucks, and driving between pits, cesspools or pumping stations for inspection and maintenance. Inspection tasks are also expected to be sources of low exposure and are present for all groups. Some main characteristics with regards to exposure are:

- Pumping station personnel are close to the waste during repair and maintenance, and flush the floor of the installation if visibly dirty. Their active work with sewage is indoor and at sites with varying ventilation. Some are so small that an open door is the only ventilation.
- In the plants, work is dominated by control room work. The better they manage to run the plant, the more time they spend in the control rooms. These plants have fresh air supply ventilation without recirculation in all areas. All floors except the control rooms are kept clean by flushing. Not all days have exposed tasks other than inspection.
- Water net workers are regularly down into pits. These are usually draining into the sewage net. In a few, but increasing number of areas, they drain into storm water pipes, that are cleaner than the sewage net.
- Sewage net workers rarely enter pits, but they flush pipes by pressurized water (200 bar) directed backwards, digging through obstacles. When the water hose is extended at full length or to the next pit, the hose is drawn back mechanically while flushing, and cleans any remaining obstacles from the pipes. This releases gas trapped in the pipe or the pipe sediments. The sewage is sucked into the tank of the vacuum truck, which doesn’t recirculate the water. If sucking is needed in plants or pumping stations, it is done by sewage net workers.

Oil and grease can cause massive clogging of wastewater pipes, especially in combination with single use towels and sanitary products. A severe example was seen in Whitechapel, London in 2017 (BBC, 2017; Wikipedia, 2021). Smaller clogging may also be problematic, as the sewage backs up and spills over. Workers sometimes need to physically enter confined spaces, with little or no ventilation, to remove obstructions. Excess sewage is initially drained away, but the space will not be totally empty of sewage. All sediments, whether clogging or excess material after draining, can still release significant amounts of H_2_S. This may be life threatening to the workers, like the case reported by Barbera et al. (2016). A normal human reaction to a colleague in need of help is to jump in. However, with H_2_S in the atmosphere more people will be at perilous risk. An effective way of reducing such risk is therefore to be prepared by learning about the dangers and the proper use of the safety equipment. Working on a clogged pipe with sewage backed up upstream of the clogging, means that the workspace may at some point be flooded by sewage, and most likely also by high H_2_S concentrations. Also cleaning of pipes with high pressure water may lead to high H_2_S exposure outside the manhole. The concentration of H_2_S in the sewage is driven by “coincidence” and biological processes. Coincidence is marked with quotes: It is not coincidental, but multiple parameters are involved in the release of the gas, including temperature, presence of sediments, pH of the sewage, pressure drops and what is entering the sewage. Part of the perceived unpredictability is tied to irregular emissions into the sewer of chemical and biological waste, for example permitted emissions from the food industry.

Ventilation and design of facilities influence the H_2_S levels and risk of peak exposure. H_2_S is a heavy gas, and if the ventilation is not designed to exhaust air from the lowest points, especially in confined spaces, high exposures might occur as workers enter the environment. Due to ventilation, natural or mechanical, episodes of high exposures take the form of peaks.

This article aims to provide a detailed description of H_2_S exposure of wastewater collection and treatment workers in Trondheim Municipality by use of exposure monitoring data collected with personal protective alarm equipment over several years. This and the number of workers with wastewater related work, makes this a large and unique dataset that we expect to give new insight about exposure profiles. This may be a basis for risk assessment and preventive measures in other municipalities.

## Methods

Data for this study was collected in Trondheim Municipality, located in the Middle of Norway. With approximately 210 000 inhabitants it is the third largest city in the country. The Wastewater department operates two large sewage treatment facilities with a total capacity of 342 000 population equivalents. Both treatment facilities are in rock caverns to protect them from harsh winter climate. They are equipped with a balanced ventilation system, including exhaust at floor level and in basins. There are also three small facilities with only exhaust ventilation, 55 pumping stations (most of which are equipped with exhaust ventilation), 1700 septic tanks and cesspools (each of which is emptied at least once a year), and about 1100 km sewerage net with associated access manholes. The wastewater workers belong to three main groups: sewerage net, sewage pumping stations and sewage treatment (plant). These groups together represent the diversity of Norwegian wastewater workers. Manholes in the water distribution net (clean, drinking water) are drained into the sewerage net, or storm sewerage net if this is in place. This makes water distribution net workers also at risk of exposure to sewage gases. They are the fourth group of workers included in this study.

The equipment worn by water and wastewater workers at Trondheim is the Honeywell BW MicroClip3, which has sensors to detect H_2_S, oxygen, carbon monoxide, and explosive atmosphere; only H_2_S data were reported in this study. The detection range of the equipment is from 1.6 to 100 ppm H_2_S with a 0.1 ppm resolution. Values are registered every 15 s for up to 16 h and have wraparound storage (Honeywell, 2020). The recorded value is the instant value in the system at the time of recording, not a mean of the 15 s logging period (Haugland-Bergsagel, 2021). The equipment can give four different H_2_S alarms: two instant values, and one time weighted (8 h TWA) to compare with OEL, and one 15 min wraparound mean to compare with STEL. STEL and instant H_2_S level of 10 ppm, and H_2_S TWA of 5 ppm are used. Alarm data is stored separately. Equipment calculated OEL and STEL values below 0.1 ppm are not recorded.

Of the 80 pieces of equipment in use, 65 are for personal use of workers, most of which are used daily. This study included data from the equipment used by 60 water and wastewater workers in Trondheim. These workers opted-in to the study after they were informed of the purpose of the study and asked for consent to use their data, and how to withdraw their consent. As each piece of equipment is assigned to a specific person, data were divided by serial number into similar exposed groups (SEG) for analysis. Data is obtained by self-administered collection. Ethical approval from the regional ethical committee (REC)/institutional review board (IRB) were not needed for this part of the study, as the data of exposure are from normal work activities and therefore not subject to ethical committee approval. Internal approval from Trondheim Municipality of the study was given by the manager of the Water and Wastewater department, and all data was collected among them. The consent form was approved by the data management representative in Trondheim municipality and the local union representative. Data was anonymized by re-coding and deleting serial numbers of the personal equipment.

Five docking stations were available. From April 2016, the docking stations were set up to bump check the equipment and transfer data at every docking, and to calibrate automatically at docking after the due date (every 180 days). Calibration and bump test fails if the calibration gas bottle is empty. Status is shown on the display of both the equipment and the docking station. Internal procedure said that the equipment should be docked end-of-shift after an alarm, and at least once a week. Charging of equipment is done at a dedicated charging unit. A log code is set when turning on or off the equipment. The equipment continuously calculates TWA and STEL in the same run.

Data were extracted from the docking stations into the BW Technologies Fleet Manager II-software. Raw data were converted to excel and imported into IBM SPSS Statistics Version 26. Descriptive information in the raw data was used to categorize and aggregate data for each detected workday per person. We used a previously published algorithm (Austigard and Smedbold, 2021) adapted to the format of the exported data files, to calculate daily exposure measures of H_2_S, such as number of peaks, peak height, TWA, and duration, for each person. Multiple runs (on and off multiple times a day) are combined for the same person on the same day.

The data was assessed by censored empirical cumulative distribution plot (cen_ecdf, Data Analysis for Censored Environmental Data, Version: 1.0.2 for R). The normality assumption was tested by Kolmogorov–Smirnov and Shapiro–Wilk test on normal and log-transformed data. Within and between worker variability were assessed by logistic regression applying the presence of measurement above LOD as dependent variable and SEG and worker as independent variables. We used the Kruskal–Wallis test to test if the distribution of H_2_S TWA and maximum concentration level differed among the SEGs. We applied Bayesian proportion testing of exceedance of CV to test if they are different. The dataset was tested for autocorrelation with the sample AutoCorrelation Function (ACF) procedure. As the ACF does not have an option for adjusting the analysis based on different intervals between cases and on multiple IDs, we, for this analysis, merged the dataset with a file containing all workdays in the detected period for each individual.

## Results

Available data are from August 2015 to March 2021, with readings from 59 out of 60 included workers. The number of data points recorded per person varied from 144 to 326 581, resulting in a total 3.0 million data points, covering more than 40 000 work hours. The data spanned 7083 workdays, where the number of identified workdays per person varied from 4 to 559, with median 71.

Fifty eight workers had H_2_S exposure registered above the limit of detection (LOD), 1.6 ppm.

In total, 14 690 of the data points (0.49% of data points, 0.15% of the time) contained values above the LOD, corresponding to 61.2 h. The difference in percentages are due to a space-saving procedure in the docking/transfer procedure, allowing removal/compression of data with only “zero” readings.

Out of the 7083 work days, 1295 (18%) contained H_2_S values above the LOD, and 424 (33%) contained H_2_S values above the CV of 10 ppm. Only one workday had TWA H_2_S concentrations that exceeded the full shift OEL of 5 ppm. Fourteen workdays had TWA that exceeded 0.5 ppm (1/10 of the OEL), and these days all had H_2_S concentration that exceeded the CV of 10 ppm. Further, 14 days had peaks exceeding 50 ppm H_2_S, of which three exceeded 100 ppm. Only half (7) of the 14 measurements with the highest peaks also had TWA H_2_S concentrations exceeding 1/10 of the OEL. Table 1 presents summary statistics across the four SEGs.

**Table 1.** Descriptive statistics from hydrogen sulphide (H_2_S) measurements for the four SEGs, and as total

Variable	Similar exposed groups (SEG)				Total
	Sewerage network	Plant	Pumping stations	Water ­distribution network	
Number of persons	23	15	6	15	59
Measured workdays	1175	3852	944	1112	7083
Number of measured workdays per person (range)	5–121	57–559	44–476	4–389	4–559
Number and (%) of measured workdays with H_2_S levels above LOD	209 (18%)	820 (21%)	213 (23%)	53 (5%)	1295 (18%)
Number of measured workdays with TWA > 1/10 OEL	1	6	7	0	14
Number of measured workdays with H_2_S levels > CV	73	257	78	16	424
% of measured workdays with H_2_S levels >CV	6 %	7 %	8 %	1 %	6 %
% of measured workdays with H_2_S levels > CV, in days above LOD	35%	31%	37%	30%	33%
Mean and (median) of measured duration per day in minutes (*N*_total_ = 7083)	210 (99)	433 (387)	332 (381)	173 (47)	342 (293)

CV, ceiling value (10 ppm); LOD, level of detection (1.6 ppm); *N*_*Total*_, total number of measured workdays; TWA, time weighted average, 8 h; OEL, occupational exposure limit, 8 h (5 ppm).

**Figure 1.**
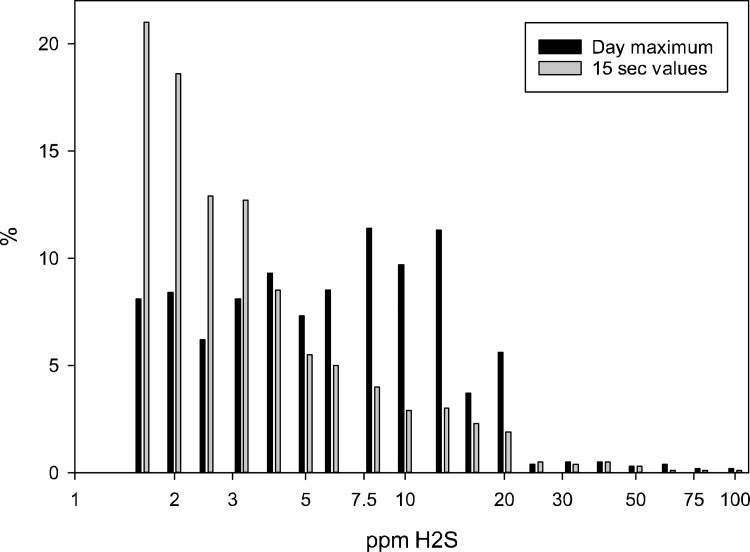
Frequency distribution of detected H2S concentrations (gray, *n* = 14 690) and daily maximum H_2_S concentrations (black, *n* = 1295). The instrument detection range is 1.6–100 ppm H2S.

In Fig. 1 the distribution of maximum levels of H_2_S per measured workday are shown, together with the total frequency of H_2_S registrations. Days with exposure below LOD are omitted. The bin at 10 ppm starts with data from 10.1 ppm, including only exceedances of CV. Right censored values are included in the bars at 100 ppm. Median of all registered 15 s H_2_S readings above LOD is 3.0 ppm, with range 1.6–101 ppm, where 101 represents all overload registrations. Mode of registrations are at 1.6 ppm H_2_S (*n* = 3079; 21%), and mode of maximum day level at 7.9 ppm H_2_S (*n* = 147; 11%).

**Figure 2.**
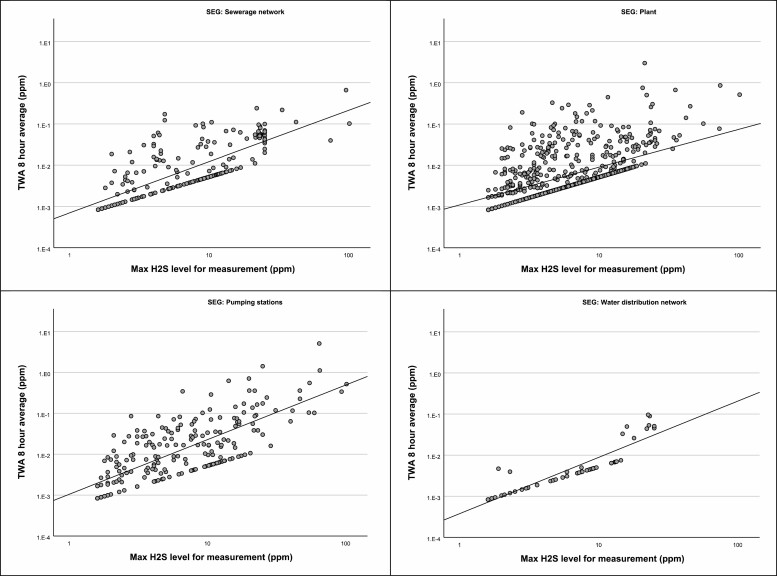
Scatterplot of maximum H2S level to time weighted average (TWA) level for measurement days with registrations above level of detection (LOD) at 1.6 ppm, with correlation lines *R*2, separated in the four SEGs: Sewerage network (*n* = 209, *R*2 = 0.57), plant (*n* = 820, *R*2 = 0.31), pumping stations (*n* = 213, *R*2 = 0.56), and Water distribution network (*n* = 53, *R*2 = 0.84).

Testing by *Q*–*Q* plot, Kolmogorov–Smirnov and Shapiro–Wilks test for normality shows that the values for TWA and maximum level of H_2_S in measured workdays are not normally or log-normally distributed, also when only positive days are evaluated. In Fig. 2 we show the scatter plots of maximum H_2_S level to TWA for measured workday in the SEGs. The line of dots at the bottom of each scatter plot represents measured days with only one registered positive data-point each. For some of these registrations this is due to the instrument not being bumped each day, and only alarm registration for the day is transferred. The *y*-axis is spanning over a magnitude of five: from 1.E−4 (0.0001 ppm) to 1.E1 (10 ppm).

**Figure 3.**
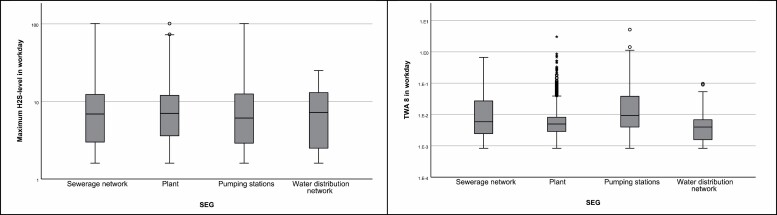
Box plot of maximum H2S level and calculated time weighted average (TWA) in workdays above level of detection (LOD) at 1.6 ppm for each similar exposed group (SEG).

In Table 2 some statistical parameters of the dataset are presented for workdays with detectable values. The large number of left censored H_2_S values in the dataset makes the median and mode of any parameter zero when calculated on all data. On only positive data, mode of TWA is <0.01, and median ≤0.01 for all SEGs and in total. In Fig. 3 we present box plots of the daily maximum H_2_S levels and TWA in workdays with detected H_2_S concentrations for each SEG.

**Table 2.** Statistics on hydrogen sulphide (H_2_S) levels in ppm for days above LOD

Variable	Similar exposed groups (SEG)				Total
	Sewerage network	Plant	Pumping stations	Water distribution network	
Maximum TWA	0.66	3.02	5.10	0.10	5.10
Median of TWA	0.01	<0.01	0.01	<0.01	0.01
Mode of TWA (*N* at mode)	<0.01 (93)	<0.01 (411)	<0.01 (65)	<0.01 (35)	<0.01 (604)
Maximum H_2_S level	>100	>100	>100	25	>100

LOD, level of detection (1.6 ppm); ppm, parts per million; TWA, time weighted average, 8 h.

The Kruskal–Wallis test rejected the null hypothesis of equal distribution across the SEGs at significance level *P* < 0.001 for H_2_S TWA concentration with and without non-detects, and for maximum H_2_S levels with non-detects. For maximum H_2_S level without non-detects, the null hypothesis was not rejected (*P* = 0.91). The proportion test of CV exceedances shows differences among all combinations of SEGs except Sewage net and Plant, evaluated by 95% credible interval. Due to the large proportion of censoring (above 80%), the within and between worker variability was evaluated by addressing the proportion of positive readings by fitting three logistic regression models (SEG + Worker, SEG and Worker). The −2 Log likelihood was used to evaluate the models, showing that the Worker model performs better than the SEG model, and that adding SEG to the Worker model does not improve the model, indicating that the within worker variability was larger than the between worker variability. The −2 Log likelihood values were 6285 vs 6523 (Chi-square test, *P* < 0.001).

Autocorrelation from one workday to the next (Lag 1) is highest (8.8% for maximum H_2_S, 3.8% for TWA) when analyzed on all workdays in the period (*n* = 83 554) and 0 imputed on days without recordings. It decreases to 5.2% and 3.4% respectively, for detected days (*n* = 7083) when detected days below LOD are imputed with 0. When run on only days above LOD and missing on the rest, the values are 3.4% and 2.6% (*n* = 1295).

Most days where H_2_S was detected have only 1 peak, as detected by the algorithm (Austigard and Smedbold, 2021). Table 3 summarizes the number of peaks observed of different sizes by SEG and in total. The maximum number of peaks found in a single day for one person was 54, of which 8 were above 10 ppm. Overall, the median number of peaks per day is 1. Pumping stations have an overall median of 2 peaks per day.

**Table 3.** Maximum number of peaks per person in different intervals of peak height of H_2_S, given for different SEGs and in total

Concentration interval	Similar exposed groups (SEG)				Total
	Sewerage network	Plant	Pumping stations	Water distribution network	
Up to 5.0 ppm	29 (1)	45 (0)	20 (1)	3 (0)	45 (1)
5.1–10 ppm	7 (0)	17(0)	9(0)	1 (0)	17(0)
Above 10 ppm	5 (0)	8 (0)	14 (0)	4 (0)	14 (0)
All peaks	35 (1)	54 (1)	28 (2)	4 (1)	54 (1)

Numbers in parentheses are median.

H_2_S, hydrogen sulphide; ppm, parts per million.

## Discussion

The results show TWA values of H_2_S well below 1/10 of OEL. High TWA levels can occur, but are so unusual that a normal campaign-based exposure monitoring program cannot be expected to detect them. About 6% (424/7083) of all measurements exceeded the CV for these data, originating from self-administrated exposure monitoring over a long period of time, corresponding to 33% of the workdays where H_2_S was detected. This implies that when focusing on the TWA, H_2_S seems to be well controlled, but we document that there still are a high number of potential hazardous peaks.

The presence of days above LOD differs between the SEGs, but when looking at only these days, the presence of H_2_S peaks exceeding the CV has a quite uniform distribution of approximately ⅓rd for all SEGs. The Kruskal–Wallis test, proportion test and Bayesian statistics all document from different angles that the amount of non-detects and days without exposure is an important factor to understand the differences of H_2_S exposure between the SEGs. Within worker variability dominates and is explained by variability in tasks for the individuals, including location, conditions in the sewage, weather conditions, and individual work practice. All handling of sewage can give high exposure, and this lowers the variability of peak exposure between groups. The total evaluation of SEG descriptions and measurements warrant keeping them as separate reporting groups.

Exceedance of the CV in 6% of cases is significant, but nevertheless, small enough to be missed in preliminary test measurements (D’Errico et al., 2022). We tested this by running 100 random draws of 3 days of H_2_S results, and found that statistically, an exceedance would be observed in only 18% of attempts when sampling over three independent days. Exceedance of the CV is rarely reported to the Norwegian EXPO database (Østrem, 2014), as the main input is air sampling results obtained by laboratory analysis, rather than data from direct reading instruments. Short-term exposure levels, and especially peak levels, are therefore rarely available for regulators and researchers. Our dataset shows that almost every violation for wastewater related work of the H_2_S exposure regulation is solely based on the CV, and that this can happen several times a day. The findings suggest that use of TWA or STEL as the only evaluation criterion for H_2_S exposure does not give sufficient protection to the workers. This must be addressed when evaluating the regulations and the health consequences for the workers. The average concentrations are low at wastewater work if adequate ventilation is installed, as peaks quickly are diluted. In earlier measurements (Austigard, 2018) we saw that high peaks are very rare at well ventilated areas, such as plants, without active sewage work, including disturbances of the sediments. The scatterplots in Fig. 2 and box-plot of maximum H_2_S levels in Fig. 3 shows the potential for exposure in different SEGs that can be detected with personal alarm equipment. These results are considered useful for other wastewater installations, as they contain data from highly exposed tasks that are uncommon, and therefore often not recorded.

The data show that drinking water workers have higher H_2_S peaks than expected, even though they are far less exposed than wastewater workers. The H_2_S exposure among drinking water workers is suspected to be caused by the drain connection between water distribution net manholes and combined sewerage and/or storm water pipes. The way water distribution net workers talk about their job, they are more frequently down into manholes than sewerage net workers. While the procedure for entering manholes requires a sensor to be hoisted down in the manhole to detect exposure levels before entering, the available data do not tell whether this is done, and observations show shortcuts. In the sewage treatment plants dedicated portable sensors are used for this detection. Nevertheless, the measured values show the potential for exposure.

The data used in this study goes back to 2015, a period of 72 600 working days (5.5 years × 220 working days/person, year × 60 persons). In total, 7083 working days were retrieved, corresponding to nearly 10% of workdays in the period, and approximately 33% of maximum expected number of detected days at minimum docking practice of once a week (mean of 1.5 day/person, week recorded). Sick leave, education days and irregular hours compensation days also reduce the possible number of workdays in this study. In addition, there have been periods without functional docking stations at each site, and reports of equipment that have not been fully docked. If an alarm sounds multiple days a week, this should cause more days to be recorded if they follow the procedure of docking after an alarm.

Duration of measurements indicated that the equipment was used differently at sewerage net and water distribution net compared to plant and pumping stations (see Table 1), with shorter duration of sampling among water distribution net workers (mean 173 min) and sewage collection net workers (mean 210 min), than the two other groups. This may be caused by turning the equipment off when driving. The same pattern was seen for days with detected H_2_S-levels. There is an increased risk of prolonged exposure if the sensors are not used during potential exposed work, as exposure is so unpredictable that the alarm sensor is essential for safety. Also, a potential exceedance of TWA may not be warned, as the equipment starts a new evaluation to OEL and STEL each time it is turned on. Workers report that they sometimes shut it off because of the annoying repetitive alarm, especially when wearing required PPE. Turning the equipment off during active work could be prevented with equipment that lets the user put off the sound alarm for a period, say 10 min. The equipment must, nevertheless, sound a new alarm if a higher exposure level or another alarm category requiring a stricter PPE level is detected during this period as the different alarm categories correspond to different evaluation endpoints of health effects and in the regulations.

The data in this study were collected by the workers themselves, in contrast to a monitoring program where occupational hygienists do the collection. Liljelind et al. (2001) demonstrated that self-administrated exposure (SAE) data do not differ in result from expert collection if the equipment is easy to operate. We can therefore trust that these data give a good picture of the exposure, and make individual exposure profiles possible. The workers will still need expert help for the interpretation of data and follow up, to ensure preventive actions and continued SAE monitoring, as collection easily declines if no information comes in return (Pettersson-Strömbäck et al., 2008).

Scrutiny of data revealed that some of the data were compressed e.g. most zero readings are deleted. Re-import of the oldest data into the software in full format was not possible, as it was overwritten at the docking stations. To control if this affected the calculation of duration and exposure, some data were imported in both full and compressed format, and then analyzed. This showed that the calculated values were not affected.

In total, 61.2 h of exposure above the LOD at 1.6 ppm was recorded. Assuming all exposed time for the 7083 days are measured, this corresponds to 0.15% of the total work time. In the previously presented expert collection among wastewater workers (Austigard, 2018; Austigard and Smedbold, 2021), 4.8% of the time were above the LOD (16 h out of 331 h), with LOD 0.1 ppm. By re-assessment of the 2018/2021 dataset we found it to contain 0.8% above 1.6 ppm (17% of all data points above 0.1 ppm). Applying this fraction (17%; 1/6th) to the SAE dataset reported here, a LOD of 0.1 ppm H_2_S would have given six times more exposed time. This would still however, be less than 1% of the total work time. The difference between 4.8 and 1% reflects that the campaign measurements focused on measuring exposed time (Austigard, 2018), while the SAE measurements in the new dataset presented here reflected both exposed and unexposed work hours.

The data presented were recorded with a 15 s interval. Logging every second, to get a closer approximation to the true TWA value, would not have met the need of the users to store at least a full day (Honeywell, 2020, p 63). The T90 time of the sensor is 10–13 s, so it does not seem relevant to shorten the recording time interval. In the analysis, the recorded data is therefore handled as if it is a mean value of the time interval. It also means that the instruments are not contributing to autocorrelation in the dataset between work tasks. Regarding the autocorrelation analysis between days, the imputed undetected workdays per year do not contain Saturdays, Sundays and public holidays but do include vacation days (5–6 weeks per person based on age). The number of days in the maximum autocorrelation test therefore contain more days than the calculation of possible workdays. The autocorrelation found is maximum 8.8% and is due to the large number of zero values. For days with readings above LOD it is 3.4% for maximum H_2_S level and 2.6% for TWA level. Our evaluation is therefore that the autocorrelation does not influence the assessment of the data.

In our tables and figures, values for median TWA and range are presented. This is due to the fact that our data is neither normal nor log-normal distributed. A log-probability plot of data above LOD shows that this seems to be due to deviations near the endpoints. This can be due to both left and right censoring of data, as mentioned. For comparison the mean and SD assuming normal or log-normal distribution are presented in Supplementary Table S1 (available at *Annals of Work Exposures and Health*). These data underline that TWA is not useful for this kind of data where the exposure is due to peaks, and the main health risk is due to acute high exposure, rather than lower long-term exposure.

For statistics on exceedance of CV, this should at least be a binary (“yes”/“no”) evaluation, together with maximum peak level. Intensity, or number of peaks, gives additional value, but is not critical for the main evaluation of compliance. For the protection of the workers, one approach is to always use a direct reading instrument, another is to always use a full-face mask. The second will not comply with thoughts and regulation on hierarchy of controls if it stands alone.

In a controlled work situation, it could be assumed that by taking more measurements the calculated uncertainty decreases. In our dataset, we see that the range is very large compared to the median, and a high number of measurements is likely to capture more of the variation in the exposure. Our high number of measurements is therefore essential to capture the high within worker variability and multimodality that is due to the range of tasks for each worker, and to the unpredictable peaks in H_2_S exposure. Redefining the SEGs would not change this.

An alternative approach to analyse and report data from direct reading instruments may be using an exposure index as suggested in an earlier publication (Austigard, 2018). There we show that in days above CV, levels below present LOD have little influence on the total index value. In this index the present exposure pattern, e.g. number of peaks, height and duration of peaks, together with the undetected background level are weighted together. The daily H_2_S max readings reported in this article is only one dimension covered by this index.

The censoring pattern reported in this study needs further study. The high percentage of censoring (>80%) within a day with detectables, and days with exposed work where all readings are below the LOD, are likely to underestimate the TWA values. This can be illustrated by calculated TWA values on expected exposed workdays, some of which are below the expected residential background level (ATSDR, 2016). A Bayesian calculation of value for non-detects is an option, but we will need some more information on activity to set the priors.

### Limitations and transferability

The recordings are left censored to 1.6 ppm, and right censored from 100 ppm. Range 1.6–100 ppm are quite usual for alarm equipment, but occupational hygienists learn that evaluating down to at least 1/10 of OEL is best practice. This equipment does not allow better than 3/10 of OEL, so low exposed work goes undetected. On the other hand, the acute peak levels are well documented by the equipment.

Workers sometimes report incidents of feeling ill without having docked the equipment to transfer data, even though the alarm equipment was used during work. It is therefore assumed that some highly exposed incidents, possibly in the right censored area, are not recorded. A decline in the number of recordings was observed from 2020, corresponding to the period of the Coronavirus disease (COVID-19) pandemic. During this period, not all workers were regularly at the sites of docking stations. In addition, there was a breakdown in one of the docking stations, and it took some months before it was replaced. This demonstrated that easy transfer is essential to be able to collect data, and complies with earlier findings (Pettersson-Strömbäck et al., 2008). However, the data collected represent all parts of the year for all SEGs. The observation of compliance of use shows that workers are better at using the equipment than of docking it. At present, the docking is necessary to make the data available. Solutions that do not demand physical docking will increase the transfer of data. Nevertheless: the 10% of days found gives far more data than campaign-based measurements made earlier. In numbers the documented days are so many, and the collection method is not biased to find 0 days, that this is representative data for evaluation, also for an unpredictable gas such as H_2_S. In total this underlines the need for continuous monitoring.

Our findings in H_2_S peak structure are transferable to other places and other sources of H_2_S, but workplaces with low ventilation rates must expect higher background H_2_S levels and longer duration of peaks. This is the case in some enclosed areas, such as older wastewater facilities and in many livestock buildings. In some settings, such as oil and gas installations, the potential for H_2_S exposure is much higher, increasing the risk of extremely high peak and prolonged exposure in the facility.

## Conclusions

Systematic collection of monitoring data from alarm equipment gives a good description of the potential maximum exposure level, but right censoring is observed. The study found that among water and wastewater workers in a large municipality in Norway, the frequency of peaks above CV is higher than expected from other sources of data, for example in the Norwegian EXPO database.

The measured exposure concentrations show that wastewater workers can be exposed to extreme H_2_S peak concentrations, while 8-h TWA exposure concentrations are well below the OEL. An ordinary preliminary measurement program over three days to evaluate further measures is unlikely to find an exceedance of 1/10 of OEL, and have an 82% probability of not finding exceedance of the CV. Without additional information, this finding will lead to the misconception that further measurements are unnecessary.

Concentrations of H_2_S at or above 100 ppm, the level of immediate danger to life and health determined by NIOSH (2019), were documented in this study. The toxicity and exposure profile of H_2_S makes continuous monitoring of exposure levels necessary for alarm purposes. This is possible today, as alarm equipment has become more available and has sensors of quality equalling measurement equipment. The boundaries of the equipment must be considered, but it is time and cost efficient to collect already available data for exposure assessments. We advocate such use, as we find evaluation of risk by TWA alone insufficient to protect the workers, regardless of method of measurement.

## Supplementary Material

wxac065_suppl_Supplementary_Table_S1

wxac065_suppl_Supplementary_Algorithm_adjustment_description

wxac065_suppl_Supplementary_Algorithm

## Data Availability

Data and algorithms will be made available at Norwegian Centre for Research Data, NSD, (www.NSD.no). The algorithm and adaptation description will also be available online as supplementary files.
