# Supplementary material for: Risk Characteristics of Hydrogen Sulphide Exposure in Wastewater Collection and Treatment Related Occupations
Source: Ann Work Expo Health. 2022 Sep 17;67(2):216–27. doi: 10.1093/annweh/wxac065 (PMC9923040; doi:10.1093/annweh/wxac065)
Supplement: wxac065_suppl_Supplementary_Table_S1 [file wxac065_suppl_supplementary_table_s1.docx]

Table S1: Statistics on hydrogen sulphide (H_2_S) levels in ppm (for days above LOD if other is not stated), and assuming normal or lognormal distribution.

| **Variable** | **Similar exposed groups (SEG)** | | | | **Total** |
| --- | --- | --- | --- | --- | --- |
|  | **Sewerage network** | **Plant** | **Pumping stations** | **Water distribution network** |  |
| Mean of TWA (SD) | 0.02 (0.06) | 0.02 (0.12) | 0.08 (0.38) | 0.01 (0.02) | 0.03 (0.18) |
| Mean of TWA of all days, (SD) | <0.01 (0.03) | <0.01 (0.06) | 0.02 (0.18) | <0.01 (0.01) | 0.01 (0.08) |
| LOD: Level of detection (1.6 ppm). ppm: parts per million. SD: Standard deviation.  TWA: Time weighted average, 8 hour. | | | | | |
