# Supplementary material for: Risk Characteristics of Hydrogen Sulphide Exposure in Wastewater Collection and Treatment Related Occupations
Source: Ann Work Expo Health. 2022 Sep 17;67(2):216–27. doi: 10.1093/annweh/wxac065 (PMC9923040; doi:10.1093/annweh/wxac065)
Supplement: wxac065_suppl_Supplementary_Algorithm_adjustment_description [file wxac065_suppl_supplementary_algorithm_adjustment_description.docx]

Algorithm adjustment description - Risk characteristics of hydrogen sulphide exposure in wastewater collection and treatment related occupations

Åse Dalseth Austigard ^1, 3^ Hans Thore Smedbold ^2,4^ Kristin von Hirsch Svendsen ^1^

^1^ Department of Industrial Economics and Technology Management, NTNU - Norwegian University of Science and Technology, PO Box 8900, Torgarden, N-7491 Trondheim, Norway. E-mail: ase.d.austigard@ntnu.no . Tel.: +47 95263902

^2^ Department of Occupational Medicine, St Olav University Hospital, PO Box 3250, Torgarden, N-7006 Trondheim, Norway

^3^ Trondheim Municipality, Working Environment Office, PO. box 2300 Torgarden, N-7004 Trondheim, Norway. E-mail: [ase-dalseth.austigard@trondheim.kommune.no](mailto:ase-dalseth.austigard@trondheim.kommune.no)

^4^Department of Public Health and Nursing, Faculty of Medicine and Health Sciences, N-7491 Trondheim, Norway

The earlier published algorithm (Austigard, 2021) was given a supplement to adapt to the format of the exported datafiles from BW Technologies Fleet Manager II-software.

System data for PowerOn and ShutDown are used in the algorithm together with measurement readings, while alarm levels, bump test data and calibration data are not included in the calculations. For each person the recorded data from one day were given the same LogID (identifyind workdays), regardless of whether it has one or multiple runs.

We have also given an adaptation to the algorithm to handle irregular intervals, for example compressed files. We have used PowerOn- and ShutDown-codes to detect different runs, so that we also detect days where the equipment has been running over multiple days. The overwriting of data means that we also have to control for new runs by evaluating the date and the interval between data. Calculation of measurement time for each workday (LogID) is also changed to handle this and the possible presence of multiple runs.

The third adjustment is an adaptation to run the algorithm and extract data for each workday (LogID) in a separate file. This proved necessary as we met the BigData problem of amount of data. The dataset became too large for the algorithm to run effectively on one aggregated datafile from all participants. This adjustment makes it easy to later add new LogIDs to an old database. According to this, we also adjusted the algorithm to handle up to 999 tasks per person, not only 99. In this adaption we also separated out all positive log-points with H_2_S in a separate file. Incidents with detected exposure that are separated by more than 3 minutes, are counted as different tasks. The arguments around this is presented in the algorithm article  (Austigard, 2021).

Reference:

Austigard, Åse Dalseth, and Hans Thore Smedbold. 2021. "Hydrogen Sulphide (H2S) Exposure Hazard Assessment: An Algorithm for Generating Exposure Index Based on Direct Instrument Readings." *Annals of Work Exposures and Health* 66, no. 1: 6. Accessed 6/29/2021. <https://dx.doi.org/10.1093/annweh/wxab047>.
