# Supplementary material for: Risk Characteristics of Hydrogen Sulphide Exposure in Wastewater Collection and Treatment Related Occupations
Source: Ann Work Expo Health. 2022 Sep 17;67(2):216–27. doi: 10.1093/annweh/wxac065 (PMC9923040; doi:10.1093/annweh/wxac065)
Supplement: wxac065_suppl_Supplementary_Algorithm [file wxac065_suppl_supplementary_algorithm.docx]

Algorithm - Risk characteristics of hydrogen sulphide exposure in wastewater collection and treatment related occupations

Åse Dalseth Austigard ^1, 3^ Hans Thore Smedbold ^2,4^ Kristin von Hirsch Svendsen ^1^

^1^ Department of Industrial Economics and Technology Management, NTNU - Norwegian University of Science and Technology, PO Box 8900, Torgarden, N-7491 Trondheim, Norway. E-mail: ase.d.austigard@ntnu.no . Tel.: +47 95263902

^2^ Department of Occupational Medicine, St Olav University Hospital, PO Box 3250, Torgarden, N-7006 Trondheim, Norway

^3^ Trondheim Municipality, Working Environment Office, PO. box 2300 Torgarden, N-7004 Trondheim, Norway. E-mail: [ase-dalseth.austigard@trondheim.kommune.no](mailto:ase-dalseth.austigard@trondheim.kommune.no)

^4^Department of Public Health and Nursing, Faculty of Medicine and Health Sciences, N-7491 Trondheim, Norway

* Encoding: windows-1252.

*RUN INSTRUCTIONS:

*Check for correct input details in filename, line 196, and corresponding ID in line 201 and 1587.

*Run Lines 190-1297: Gets file and generates data. (1st import).

*Run Lines 1300-1592: Generates aggregated file from the generated data.

* Stores selected output for each person.

*Run Lines 127 - 166: Part B - Saves files from the 1st run.

*From 2nd run and on:

* Change input details in filename line 196 and corresponding ID in line 201 and 1587.

* Run from line 190 till end.

* Repeat untill all files are proseesed.

*The sorting error message from line 839 is controlled, and result OK.

*TIP:

*For quicker runs, "save outfile" in lines 1604-1640 can be scipped for some runs.

*It is adviced to give number to these files, corresponding to the last ID that is aggregated, to ease reopening.

*Lines 168-178 are used to reopen aggregated files if all import/ generation is not done at the same session.

*Lines 36-113 are used to extract selected dates.

*CONTENT: lines 1 - 33 .

*Part A. Lines 36 - 113 - Save selected dates in dedicated file.

*Part B. Lines 127 - 166 - Generating and storing files after 1st run.

*Part C. Lines 169 - 179 - Retain stored, aggregated files.

*Part D. Lines 190 - 206 - Import of data.

*Part E. Lines 209 - 283 - Adaption to Fleet Manager II format in export.

*Part F. Lines 286 - 590 - Automatized detection of different measurements and runs.Cal and bump data.

*Part G. Lines 593 - 731 - Test and tag censoring, negative values ++.

*Part H. Lines 734 - 984 - Algorithm preparation data:

*Part I. Lines 987 -1135 - Algorithm generation:

*Part J: Lines 1138 -1297 - Other calculations:

*Part K. Lines 1300 - End - Aggregating data to separate files. Print parts of output.

* Part A. Save selected dates in dedicated file.

DATASET ACTIVATE Aggr_Index_all WINDOW=FRONT.

COMPUTE Date_selected =0.

IF ((Year=2019) and (Month=2) and (Day=25 or Day=26 or Day=27 or Day=28)) Date_selected =1.

IF ((Year=2019) and (Month=3) and (Day=1 or Day=2 or Day=3)) Date_selected =1.

IF ((Year=2019) and (Month=3) and (Day=4 or Day=5 or Day=6 or Day=7 or Day=8 or Day=9 or Day=10)) Date_selected =1.

IF ((Year=2019) and (Month=6) and (Day=3 or Day=4 or Day=5 or Day=6 or Day=7 or Day=8 or Day=9)) Date_selected =1.

IF ((Year=2019) and (Month=6) and (Day=10 or Day=11 or Day=12 or Day=13 or Day=14 or Day=15 or Day=16 or Day=17)) Date_selected =1.

IF ((Year=2019) and (Month=9) and (Day=9 or Day=10 or Day=11 or Day=12 or Day=13 or Day=14 or Day=15)) Date_selected =1.

IF ((Year=2019) and (Month=9) and (Day=16 or Day=17 or Day=18 or Day=19 or Day=20 or Day=21 or Day=22)) Date_selected =1.

EXECUTE.

Compute YearSelected =0.

If Year =2019 YearSelected =1.

EXECUTE.

Sort cases by YearSelected (D) Date_selected (A) ID(A).

Filter by YearSelected.

Split file by ID.

CREATE

/SelectLogID = CSUM(Diff).

EXECUTE.

*Gives error message on sorting. Is OK.

If YearSelected =0 SelectLogID =0.

EXECUTE.

VARIABLE LABELS SelectLogID 'Selected and unselectede person-day-LogID in selected year, separate consecutive lists'.

FORMATS Date_selected(F1.0) YearSelected(F1.0) SelectLogID(F3.0).

FILTER OFF.

USE ALL.

DATASET COPY Aggr_index_selected.

DATASET ACTIVATE Aggr_index_selected.

SELECT IF (Date_selected =1).

EXECUTE.

SAVE OUTFILE='Filepath\aggr_index_selected.sav'

/COMPRESSED.

DATASET COPY Aggr_index_Otherdays.

DATASET ACTIVATE Aggr_index_Otherdays.

FILTER OFF.

USE ALL.

SELECT IF (Date_selected =0).

EXECUTE.

SAVE OUTFILE='Filepath\Aggr_index_Otherdays.sav'

/COMPRESSED.

DATASET CLOSE Aggr_index_selected.

DATASET CLOSE Aggr_index_otherdays.

DATASET ACTIVATE Positive_other_Readings WINDOW=FRONT.

COMPUTE Date_selected =0.

IF ((Year=2019) and (Month=2) and (Day=25 or Day=26 or Day=27 or Day=28)) Date_selected =1.

IF ((Year=2019) and (Month=3) and (Day=1 or Day=2 or Day=3)) Date_selected =1.

IF ((Year=2019) and (Month=3) and (Day=4 or Day=5 or Day=6 or Day=7 or Day=8 or Day=9 or Day=10)) Date_selected =1.

IF ((Year=2019) and (Month=6) and (Day=3 or Day=4 or Day=5 or Day=6 or Day=7 or Day=8 or Day=9)) Date_selected =1.

IF ((Year=2019) and (Month=6) and (Day=10 or Day=11 or Day=12 or Day=13 or Day=14 or Day=15 or Day=16 or Day=17)) Date_selected =1.

IF ((Year=2019) and (Month=9) and (Day=9 or Day=10 or Day=11 or Day=12 or Day=13 or Day=14 or Day=15)) Date_selected =1.

IF ((Year=2019) and (Month=9) and (Day=16 or Day=17 or Day=18 or Day=19 or Day=20 or Day=21 or Day=22)) Date_selected =1.

EXECUTE.

DATASET COPY Positive_other_Readings_selected.

DATASET ACTIVATE Positive_other_Readings_selected.

FILTER OFF.

USE ALL.

SELECT IF (Date_selected =1).

EXECUTE.

SAVE OUTFILE='Filepath\Positive_other_Readings_selected.sav'

/COMPRESSED.

DATASET CLOSE Positive_other_Readings_selected.

DATASET ACTIVATE Positive_Readings WINDOW=FRONT.

COMPUTE Date_selected =0.

IF ((Year=2019) and (Month=2) and (Day=25 or Day=26 or Day=27 or Day=28)) Date_selected =1.

IF ((Year=2019) and (Month=3) and (Day=1 or Day=2 or Day=3)) Date_selected =1.

IF ((Year=2019) and (Month=3) and (Day=4 or Day=5 or Day=6 or Day=7 or Day=8 or Day=9 or Day=10)) Date_selected =1.

IF ((Year=2019) and (Month=6) and (Day=3 or Day=4 or Day=5 or Day=6 or Day=7 or Day=8 or Day=9)) Date_selected =1.

IF ((Year=2019) and (Month=6) and (Day=10 or Day=11 or Day=12 or Day=13 or Day=14 or Day=15 or Day=16 or Day=17)) Date_selected =1.

IF ((Year=2019) and (Month=9) and (Day=9 or Day=10 or Day=11 or Day=12 or Day=13 or Day=14 or Day=15)) Date_selected =1.

IF ((Year=2019) and (Month=9) and (Day=16 or Day=17 or Day=18 or Day=19 or Day=20 or Day=21 or Day=22)) Date_selected =1.

EXECUTE.

DATASET COPY Positive_Readings_selected.

DATASET ACTIVATE Positive_Readings_selected.

FILTER OFF.

USE ALL.

SELECT IF (Date_selected =1).

EXECUTE.

SAVE OUTFILE='Filepath\Positive_Readings_selected.sav'

/COMPRESSED.

DATASET CLOSE Positive_Readings_selected.

*End part A.

*Part B.

*/Generating files after 1st run:.

*B.1. Aggregated data.

DATASET ACTIVATE AggrIndex0.

SAVE OUTFILE='Filepath\Aggr_Index_all01.sav'

/COMPRESSED.

DATASET NAME Aggr_Index_all WINDOW=FRONT.

*B.2.Positive readings of H2S.

DATASET ACTIVATE DataSet1.

DATASET COPY Positive_Readings0.

DATASET ACTIVATE Positive_Readings0.

FILTER OFF.

USE ALL.

SELECT IF (H2S_positive =1).

EXECUTE.

SAVE OUTFILE='Filepath\Positive_Readings01.sav'

/COMPRESSED.

DATASET ACTIVATE Positive_Readings0 WINDOW=FRONT.

DATASET NAME Positive_Readings WINDOW=FRONT.

*B.3. Positive readings regardless of type.

DATASET ACTIVATE DataSet1.

DATASET COPY Positive_other_Readings0.

DATASET ACTIVATE Positive_other_Readings0.

FILTER OFF.

USE ALL.

SELECT IF (Sensors_data =1).

EXECUTE.

SAVE OUTFILE='Filepath\Positive_other_Readings01.sav'

/COMPRESSED.

DATASET ACTIVATE Positive_other_Readings0 WINDOW=FRONT.

DATASET NAME Positive_other_Readings WINDOW=FRONT.

DATASET CLOSE Dataset1.

*End Part B - generating files after 1st run.

*Part C: If you have to close an go on later. Number at end is last run ID:.

GET

FILE='Filepath\Aggr_Index_all01.sav'.

DATASET NAME Aggr_Index_all WINDOW=FRONT.

GET

FILE='Filepath\Positive_Readings01.sav'.

DATASET NAME Positive_Readings WINDOW=FRONT.

GET

FILE='Filepath\Positive_other_Readings01.sav'.

DATASET NAME Positive_other_Readings WINDOW=FRONT.

*End part C - Retain stored, aggregated files.

*Part D: *** Start Import ****

**********************************

*** Getting raw data ******************************************************.

* </sheet=name 'name'> must be included in line following "/file" in the programe if multiple sheets are present in file.

GET DATA

/TYPE=XLSX

/FILE='Filepath\Filename_ID.xlsx'

/CELLRANGE=FULL

/READNAMES=ON

/DATATYPEMIN PERCENTAGE=95.0

/HIDDEN IGNORE=YES.

DATASET NAME DataSet1 WINDOW=FRONT.

Compute ID=060.

FORMATS ID(F3.0).

ALTER TYPE SerialNumber(A13) LogType(A12) LogTime(DATETIME40) Status(A20) Bump(A5) H2SReading(F5.1) H2SSTEL(F5.1) H2STWA(F5.1) COReading(F5.1)

COSTEL(F5.1) COTWA(F5.1) O2Reading(F5.1) LELReading(F7.1) H2SStatus(A15) COStatus(A15) O2Status(A15) LELStatus(A15) UnitStatus(A15) UnitOptions(A15)

Language(A10) GasConfiguration(A20) CalInterval(F5) BumpInterval(F5) HighAlarm(F5.1) LowAlarm(F7.1) TWAAlarm(F5.1) STELAlarm(F5.1) STELPeriod(F5).

Filter off.

Use all.

SORT CASES by ID SerialNumber LogTime.

EXECUTE.

*End Part D - Import.

*Part E:. Adaption to Fleet Manager II format in export:.

*E.1. Cathegorizing and taging of data.

COMPUTE h2s=H2SReading.

COMPUTE Readings = 0.

IF (LogType = 'Readings') Readings =1.

IF (Bump = 'Yes') Readings =0.

IF (Status = 'Auto-Zeroing') Readings = 0.

COMPUTE BumpTest=0.

IF (Bump = 'Yes') BumpTest =1.

COMPUTE PowerUp = 0.

IF (LogType = 'Power Up') PowerUp =1.

IF (LogType = 'Power Up') Readings =1.

Execute.

COMPUTE Shutdown = 0.

IF (LogType = 'Shutdown') Shutdown =1.

IF (LogType = 'Shutdown') Readings =1.

COMPUTE Calibrating = 0.

IF ((H2SReading = 25) and (COReading = 100)) Calibrating =1.

IF ((LELReading = 50) and (COReading = 100)) Calibrating =1.

IF ((H2SReading = 25) and (LELReading = 100)) Calibrating =1.

IF (Status = 'Calibrating') Calibrating = 1.

IF (Status = 'Calibrating') Readings = 0.

IF (Calibrating = 1) Readings =0.

EXECUTE.

*E.2. If long-string timestamp: * Date and Time Wizard: Year Month Day.

COMPUTE Year=XDATE.YEAR(LogTime).

COMPUTE Month=XDATE.MONTH(LogTime).

COMPUTE Day=XDATE.MDAY(LogTime).

COMPUTE Time=LogTime.

EXECUTE.

*E.3. Other data than H2S from sensors:

**marks measurement =1 if any positive data present.

DO IF Readings=1.

+COMPUTE Sensors_data =0.

+IF ((H2SReading<>0) or (COReading <>0) or (O2Reading <>20.9) or (LELReading <>0)) Sensors_data =1.

+IF Readings =0 Sensors_data =0.

END IF.

EXECUTE.

*Marks wich sensors have positive data. (H2S in next part).

COMPUTE CO_positive=0.

IF (COReading > 0) CO_positive=1.

IF Readings =0 CO_positive=0.

EXECUTE.

COMPUTE LEL_positive=0.

IF (LELReading > 0) LEL_positive=1.

IF Readings =0 LEL_positive=0.

EXECUTE.

COMPUTE O2_not_20.9=0.

IF (O2Reading <> 20.9) O2_not_20.9=1.

IF Readings =0 O2_not_20.9=0.

EXECUTE.

VARIABLE LABELS H2S 'Registred H2S level'

/Readings '1=reading'

/BumpTest '1=bumptest reading'

/PowerUp '1= PowerUp'

/ShutDown '1 = Shut down'

/Calibrating '1 =calibration reading'

/Year "Year"

/Month "Month"

/Day "Day"

/Time 'Timestamp YMDHMS'

/Sensors_data 'Tag: 1= Detected data (H2S, CO, LEL and/or O2)'

/CO_positive 'CO level detected'

/LEL_positive 'LEL level detected'

/O2_not_20.9 'O2 level different from 20.9'.

VARIABLE LEVEL Year(SCALE) Month(SCALE) Day(SCALE).

FORMATS Readings (f1.0) BumpTest(F1.0) PowerUp (f1.0) ShutDown (f1.0) Calibrating (f1.0)

/Year(F4.0) Month(F2.0) Day(F2.0) Time(YMDHMS)

/Sensors_data(F1.0) CO_positive(f1.0) LEL_positive(f1.0) O2_not_20.9(f1.0).

VARIABLE WIDTH ID(6) H2S(8) Readings(6) BumpTest(6) PowerUp(6) ShutDown(6) Calibrating(6)

/Year(6) Month(6) Day(6) Time(12)

/Sensors_data(8) CO_positive(6) LEL_positive(6) O2_not_20.9(6).

EXECUTE.

*End of part E.

*Part F: Automatized detection of different runs and measurements.

*F.1. Differensiating runs.

*if system data on same time as reading, the Reading will come first. Securing system data after PowerUp.

SORT CASES by ID(A) SerialNumber(A) Time(A) Readings(D) PowerUp (D) ShutDown (A).

EXECUTE.

*SPLIT FILE SEPARATE BY ID. /*only nessesary if multiple ID in single file.

*EXECUTE.

CREATE YearLag = LAG(Year, 1).

CREATE MonthLag = LAG (Month, 1).

CREATE DayLag = LAG(Day, 1).

EXECUTE.

COMPUTE YearDiff = YearLag - Year.

COMPUTE MonthDiff = MonthLag - Month.

COMPUTE DayDiff = DayLag - Day.

EXECUTE.

COMPUTE Diff = ABS(YearDiff) + ABS(MonthDiff) + ABS(DayDiff).

EXECUTE.

IF Diff > 0 Diff =1.

EXECUTE.

*/now it is sysmis on "Diff" 1st row in 1st log.

Create LagShutDown = LAG(ShutDown, 1).

Execute.

COMPUTE RunDiff = 0.

COMPUTE RunDiff = LagShutDown + PowerUp.

IF sysmis(Diff) RunDiff =1.

EXECUTE.

IF RunDiff >1 RunDiff=1.

Execute.

*In case of consecutive ShutDown (one case observed; 1 sec in between):.

If (LagShutDown=1 and ShutDown =1) Rundiff=0.

Execute.

*Guaranteed runtime om instrument is 12 hours, but experience says up to 3 work days. Corrects for overvriting data before new dockong.

If (((abs(Yeardiff)) +(Abs(Monthdiff)) + (ABS(daydiff)))>4) Rundiff=1.

EXECUTE.

Compute TotDiff =0.

Compute TotDiff= diff+ rundiff.

IF sysmis(Diff) TotDiff =1.

If Totdiff>1 TotDiff=1.

EXECUTE.

CREATE

/Run=CSUM(RunDiff).

EXECUTE.

AGGREGATE

/OUTFILE=* MODE=ADDVARIABLES OVERWRITE = yes

/BREAK=Run

/RunTime_min =min(Time)

/RunTime_max = max(Time)

/RunreadingN0 = sum(Readings)

/RunN0 = N.

EXECUTE.

Do if rundiff=1.

+Compute RunreadingN =0.

+Compute RunreadingN= RunreadingN0.

+Compute RunN =0.

+Compute RunN=RunN0.

End if.

EXECUTE.

DELETE VARIABLES RunN0 RunReadingN0.

Execute.

*Check for longrun.

Compute runtime0 =0.

Compute runtime0 =(RunTime_Max - RunTime_Min)/60.

EXECUTE.

Compute LongRun =0. /*Longrun = more than 11 hours.

If RunTime0>660 LongRun=1.

Execute.

*Check for passing midnight in run:.

Compute PM_AM0 =0.

If (Diff=1 and rundiff=0) PM_AM0 =1.

EXECUTE.

*Spesial case for 1st row:.

IF (missing(diff) and RunreadingN =1) PM_AM0 =1.

Execute.

AGGREGATE

/OUTFILE=* MODE=ADDVARIABLES OVERWRITE = yes

/BREAK=Run

/PM_AM =max(PM_AM0)

/PM_AM_RunSum = sum(PM_AM0).

EXECUTE.

*F.2. Differenciating measurement days.

IF sysmis(Diff) Diff =1. /*Gives count to first row in first log.

If Diff =1 Readings =1. /*Secures the counting of ID ++.

EXECUTE.

*SPLIT FILE OFF. /*only nessesary if multiple ID in single file.

COMPUTE LOGID0 = 0.

CREATE

/LOGID0=CSUM(Diff).

EXECUTE.

AGGREGATE

/OUTFILE=* MODE=ADDVARIABLES OVERWRITE = yes

/BREAK=Logid0

/Diffsum = sum(totDiff).

EXECUTE.

IF ID <> 0 Lnr_all = $casenum. /*Will not give number to those without signed inclusion (ID=0).

EXECUTE.

AGGREGATE

/OUTFILE=* MODE=ADDVARIABLES OVERWRITE = yes

/BREAK=ID

/N_LnrID_sum=max(Lnr_all).

EXECUTE.

AGGREGATE

/OUTFILE=* MODE=ADDVARIABLES OVERWRITE = yes

/BREAK=LOGID0

/N_logid0 = N

/N_PowerUp = sum(PowerUp)

/N_ShutDown = sum(ShutDown)

/BumpPresent = max(bumptest)

/CalibrationPresent = Max(Calibrating).

EXECUTE.

AGGREGATE

/OUTFILE=* MODE=ADDVARIABLES OVERWRITE = yes

/BREAK=run

/Bump_inRun = max(bumptest)

/Cal_inRun = Max(Calibrating).

EXECUTE.

COMPUTE RunMin_hhmm =0.

COMPUTE RunMin_hhmm = (XDATE.TIME(RunTime_min)/3600).

COMPUTE RunMax_hhmm =0.

COMPUTE RunMax_hhmm = (XDATE.TIME(RunTime_max)/3600).

COMPUTE Time_hhmm = 0.

COMPUTE Time_hhmm =(XDATE.TIME(Time)/3600).

Compute CorrTime_min = RunMin_hhmm.

Compute CorrTime_max = RunMax_hhmm.

Execute.

*Daynumber i year.

COMPUTE RunMin_day =XDATE.JDAY(RunTime_min).

COMPUTE RunMax_day =XDATE.JDAY(RunTime_max).

Compute Time_day = XDATE.JDAY(Time).

EXECUTE.

*correction for pasing midnight:.

If (RunMin_day< Time_day) CorrTime_min =0.001.

If (RunMax_day> Time_day) CorrTime_max =23.999.

EXECUTE.

*Setting 24 hour logtime on singel readings without system data:.

If ((N_Logid0 =1 or RunreadingN =1) and diffsum =1 and Readings =1 and PowerUp =0) CorrTime_min =0.0001.

If ((N_Logid0 =1 or RunreadingN =1) and diffsum =1 and Readings =1 and ShutDown=0) CorrTime_max =23.9999.

If ((N_Logid0 =1 or RunreadingN =1) and diffsum =1 and Readings =1 and PowerUp=0 and ShutDown=0) runtime0 =1440.

If ((N_Logid0 =1 or RunreadingN =1) and diffsum <1 and Readings =1 and PowerUp=0 and ShutDown=0) runtime0 =0.25.

EXECUTE.

*Avoid error message due to div by 0 if only system data: Readings corrected previously.

If (corrTime_max = CorrTime_Min) corrtime_max = (Corrtime_min+0.01).

Execute.

Compute RunLogTime0 = CorrTime_max - CorrTime_min.

EXECUTE.

DO IF Totdiff=1.

+COMPUTE RunLogTime =0.

+COMPUTE RunLogTime = RunLogTime0.

END IF.

EXECUTE.

**Time in separate periods during LogID1.

* *Excludes system data and logs less than 3 min (not expected to be measurements).

* *If RunLogTime0<0.05 Readings =0.

COMPUTE LOGID1 = LOGID0.

EXECUTE.

IF Readings = 0 LOGID1 = 0.

EXECUTE.

SORT CASES BY Readings(D) LOGID1 (A) Time (A) PowerUp (D) ShutDown(A).

IF (LOGID1 <> 0) Lnr = $casenum.

EXECUTE.

If LogID1 =0 RunLogTime=0.

EXECUTE.

SORT CASES BY LOGID1 (A) readings(D) Time (A) PowerUp (D) ShutDown(A).

EXECUTE.

AGGREGATE

/OUTFILE=* MODE=ADDVARIABLES OVERWRITE = yes

/BREAK=LOGID1

/N_logid = N

/Run_inLogID = sum(totDiff)

/Time_logID_full = sum(RunLogTime).

EXECUTE.

DO IF Diff=1.

+COMPUTE Time_LogID = Time_logID_full*60.

END IF.

EXECUTE.

*Extracting weekday. 1= sunday, 7 = saturday by default.

Compute weekday=0.

Compute weekday =XDATE.WKDAY(time).

EXECUTE.

*F.5. Calibration and bump dates information on Log.

*Sorts out bump and cal information.

SORT CASES by Lnr_all(A).

EXECUTE.

DO IF LogType ='H2S Options'.

+Compute LastH2SBump0 =0.

+Compute LastH2SCal0 = 0.

+Compute LastH2SBump0 = LastBumpDate.

+Compute LastH2SCal0 = LastCalDate.

END IF.

EXECUTE.

*Bump and cal information.

*Spreads the info of bump an cal to all lines in same LogID1-File.

*- makes it available also when system data are taken out in LogID1.

AGGREGATE

/OUTFILE=* MODE=ADDVARIABLES OVERWRITE = yes

/BREAK=run

/LastH2SBump = max(LastH2SBump0)

/LastH2SCal= max(LastH2SCal0).

EXECUTE.

*Prolongs Bump and Cal info to next run if missing. If missing in first run, it will still be missing:.

DO IF sysmis(LastH2SCal).

+Compute LastH2SCal = Lag(LastH2SCal).

+Compute LastH2SBump = lag(LastH2SBump).

+Compute BumpCalGenerated =1.

END IF.

Execute.

*Prescribes Bump and Cal info to first run if missing:.

SORT CASES by Lnr_all(D).

EXECUTE.

DO IF sysmis(LastH2SCal).

+Compute LastH2SCal = Lag(LastH2SCal).

+Compute LastH2SBump = lag(LastH2SBump).

+Compute BumpCalGenerated =2.

END IF.

Execute.

*Negative value means a later date is set as bump or calibration information.

Compute DaysfromBump=0.

Compute DaysfromCal =0.

Compute DaysfromBump =rnd((Logtime-LastH2SBump)/86400).

Compute DaysFromCal =rnd((Logtime-LastH2SCal)/86400).

EXECUTE.

*If calibration or bumb is done within the run or within the day, Daysfrombump and DaysFromCal is set to 0..

IF (bump_inRun=1 or BumpPresent=1) DaysfromBump =0.

IF (Cal_inRun=1 or CalibrationPresent =1) Daysfromcal =0.

Execute.

* If calibration i don later than bump, days from bump is replased with days from calibration:.

Do If DaysfromBump> DaysfromCal.

+ Compute DaysfromBump=daysfromCal.

+Compute BumpCalGenerated = 3.

END IF.

EXECUTE.

If (DaysFrombump<0 or DaysFromCal<0) BumpCalGenerated =4.

Execute.

If RunreadingN =0 LogID1 =0.

EXECUTE.

VARIABLE LABELS YearDiff "Different year if <>0"

/MonthDiff "Different month if <>0"

/DayDiff "Different day if <>0"

/Diff '1= next measurement'

/RunDiff '1= startpoint of new run'

/TotDiff 'diff=1 and/or Rundiff =1'

/Run 'Run-nr on ID'

/RunTime_Min 'Starttime for run'

/RunTime_Max 'EndTime for run'

/RunreadingN 'nr of readings in run'

/RunN 'Nr of logs in run'

/Runtime0 'Toral runtime (min)'

/LongRun 'Mark if run>11 h'

/PM_AM0 'Passing midnight - all in run'

/PM_AM 'Mark once - Passing midnight'

/PM_AM_RunSum 'nr of midnights in run'

/LOGID0 'std measurement nr including system status logg (CSUM(diff))'

/Lnr_all 'All consecutive line numbers'

/N_LnrID_sum 'Total number of rows on ID'

/N_Logid0 'Number of datapoints in log - incl system data'

/N_PowerUp 'Nr of PowerUps'

/N_ShutDown 'Nr of ShutDowns'

/BumpPresent '1= bump present in Log'

/CalibrationPresent '1= Cal present in Log'

/RunMin_hhmm 'Starttime run (hhmm)'

/RunMax_hhmm 'Endtime run (hhmm)'

/Time_hhmm 'Time (hhmm)'

/CorrTime_Min 'Starttime LogID without date. Corrected (hh.mm)'

/CorrTime_Max 'Endtime LogID without date. Corrected (hh.mm)'

/RunMin_day 'Day of year - min'

/RunMax_day 'Day of year - max'

/Time_day 'Day of year - present line'

/RunLogTime0 'Runtime in hhmm - all lines'

/RunLogTime 'Duration of run (hhmm)(Only if TotDiff=1)'

/LOGID1 'Measurement nr (-system logs)'

/Lnr 'Readings - Consecutive line number'

/N_Logid 'Number of datapoints in log'

/Run_inLogID 'Nr of runs in LogID1'

/Time_LogID_full 'Dur. LogID with decimals (hh.mm)'

/Time_LogID "Duration logID in min - once in LogID"

/Weekday 'Weekday, starting with 1= sunday'

/LastH2SBump0 'Date of last bump - on system line'

/LastH2SCal0 'Date of last cal - on system line'

/LastH2SBump 'Date of last bump'

/LastH2SCal 'Date of last cal'

/DaysfromBump 'Days from last bump'

/DaysFromCal 'Days from last cal'

/BumpCalGenerated 'If number: Generated bump and cal dates'.

VARIABLE LEVEL Time_logID (SCALE) Time_LogID_full (SCALE).

VALUE LABELS BumpCalGenerated

1 'date of bump and cal generated from previous log'

2 'date of bump and cal generated from next log'

3 'Days since bump days replaced with cal'

4 'Prescribed bump and/or caldate irrelevant'.

FORMATS YearDiff(F1.0) MonthDiff(F2.0) DayDiff(F2.0) Diff(F2.0) LagShutDown (F1.0)

/RunDiff(F1.0) TotDiff(F1.0) Run(F5.0) RunTime_min (YMDHMS) RunTime_max (YMDHMS) RunreadingN(F6.0)

/RunN(F4.0) runtime0(F4.0) LongRun(F1.0) PM_AM0(F1.0) PM_AM (F1.0) PM_AM_runsum (F1.0)

/LOGID0(f5.0) Lnr_all(F8.0) N_LnrID_sum (F7.0) N_Logid0(F4.0) N_PowerUp(F1.0) N_ShutDown(F1.0)

/BumpPresent(F1.0) CalibrationPresent(F1.0) CorrTime_min(F4.2) CorrTime_Max(F4.2)

/RunMin_day (F3.0) RunMax_day (F3.0) Time_day (F3.0) LOGID1(f5.0) Lnr(F8.0) N_logid(F4.0)

/Run_inLogID(F2.0) Time_LogID_full (F5.2) Time_logID(F5.0) Weekday(F1.0)

/LastH2SBump(YMDHMS) LastH2SCal(YMDHMS) BumpCalGenerated(F1.0)

/DaysfromBump(F3.0) DaysFromCal(F3.0).

VARIABLE WIDTH YearLag(6) MonthLag(6) DayLag(6) YearDiff(6) MonthDiff(6) DayDiff(6) Diff(4)

/LagShutDown(6) RunDiff(5) TotDiff(5) Run(6) RunTime_min(12) RunTime_max(12) RunreadingN(8)

/RunN(6) Runtime0(8) LongRun(6) PM_AM0(6) PM_AM(6) PM_AM_RunSum(8)

/LogID0(6) Lnr_all(9) N_LnrID_sum(9) N_LOGID0(8) N_PowerUp(8) N_ShutDown(7)

/BumpPresent(8) CalibrationPresent(8) RunMin_hhmm(8) RunMax_hhmm(8) CorrTime_min(8) CorrTime_max(8)

/RunMin_day (6) RunMax_day(6) RunLogTime(6) LogID1(6) Lnr(9) N_LOGID(6) Run_InLogID(6)

/Time_LogID_full(8) Time_logID(5) Weekday(6)

/LastH2SBump0(8) LastH2SCal0(8) LastH2SBump(12) LastH2SCal(12) LogID1(6) Lnr(9) N_LOGID(6)

/DaysFromBump(8) DaysFromCal(8).

EXECUTE.

**End of Part F.

** Part G. Test and tag different logs.

*G.1.: Negative values?. /* Evaluates negative values as neglectable;

*******Must be controlled! Possible cause: drifting or cross sensitivity.

COMPUTE new_h2s=h2s.

EXECUTE.

COMPUTE H2S_negative=0.

IF (new_h2s < 0) H2S_negative=1.

EXECUTE.

IF h2s<0 new_h2s=0.

EXECUTE.

*G.2. Marks detected level of H2S.

COMPUTE H2S_positive=0.

IF (new_h2s > 0) H2S_positive=1.

IF Readings =0 H2S_positive=0.

IF Readings =0 New_h2s =0.

EXECUTE.

*G.3 Right censord marked.

*/If other equipment: value "100" replaced with relevant upper detection limit.

COMPUTE H2S_hi =0.

IF H2S >100 H2S_hi=1.

EXECUTE.

IF (Readings=1 and H2S_hi =1) new_h2s=101.

EXECUTE.

*G.4 Duration of log interval with and without system data.

SORT CASES by Lnr_all.

SPLIT FILE SEPARATE BY LOGID0.

CREATE

/interval0=DIFF(Time 1).

COMPUTE interval0=CTIME.MINUTES(interval0).

EXECUTE.

SPLIT FILE OFF.

EXECUTE.

SORT CASES by LogID1 Lnr.

SPLIT FILE SEPARATE BY LOGID1.

CREATE

/interval1=DIFF(Time 1).

COMPUTE interval1=CTIME.MINUTES(interval1).

EXECUTE.

IF Readings =0 Interval1=0.

IF Interval0<0 Interval1=0.

If (missing(Interval1) and diff=1 and Longrun=1) Interval1 =0.25.

*Standard log interval = 15 sec (0.25) set on missings in longrun.

EXECUTE.

Create

/interval2 =lead(interval1 1).

EXECUTE.

IF (Missing(interval2) and H2S_positive =1) Interval2 = Interval1.

IF (H2S_positive) Interval2 =0.25.

EXECUTE.

IF (N_logid =1 ) Interval2 =0.25.

Execute.

SPLIT FILE OFF.

EXECUTE.

SORT CASES by Lnr.

Filter by readings.

EXECUTE.

AGGREGATE

/OUTFILE=* MODE=ADDVARIABLES OVERWRITE = yes

/BREAK=LOGID1

/Calc_dur =sum(Interval2)

/median_dur = median(Interval2)

/SD_interval2 =SD(Interval2).

EXECUTE.

*if multiple runs in LOGID1, Calc_dur will fail to give the right total time.

Filter OFF.

EXECUTE.

*G.5../Control parameter duration of measurement. Pure system logs excluded:.

*if dur_check higher than LogRunTime: expect full data, but also some intervals shorter than expected logging interval (0.25 min).

*If lower than LogRunTime: not full data.

Sort cases by Lnr.

*If your standard logging rate differs form 15 sec, you must change the 0.25 to actual value.

COMPUTE dur_check = N_logid*0.25.

COMPUTE FullData =0.

COMPUTE FullDay =0.

EXECUTE.

*Contols that more than >90% of the measurement consists of 15 s interval recordings.

IF (Dur_check > (0.9*60*time_logid_full)) Fulldata =1.

EXECUTE.

*Controls that the measurement is 4 hours or more. This is called "full day", as active wastewater work,

* in our groups, rarely exceedes 5 hours a day.

IF (Time_logID_full >3.99) FullDay =1.

EXECUTE.

IF (Readings =0) FullDay =0.

EXECUTE.

*G.6. Tag parameters.

Sort cases by Lnr.

AGGREGATE

/OUTFILE=* MODE=ADDVARIABLES OVERWRITE = yes

/BREAK=LOGID1

/H2Spresent = max(H2S_positive)

/COpresent = max(CO_positive)

/LELpresent = max(LEL_positive)

/O2deviation = max(O2_not_20.9)

/R_censored = max(H2S_hi).

EXECUTE.

VARIABLE LABELS new_h2s 'Measured level - corrected'

/H2S_negative 'Tag: 1= negative value in original data'

/H2S_positive 'Tag: 1=not a 0-value'

/H2S_hi '1=Reading above Upper detection limit'

/Interval0 'interval including system data'

/Interval2 'logging interval in min'

/Calc_dur 'Sum of interval2'

/median_dur 'Median of Interval2'

/SD_interval2 'SD of Interval2'

/Dur_check 'Expected duration if 15 sec interval'

/FullData '1= regular logging intervals'

/FullDay '1=4 hours registration or more'

/H2Spresent 'Tag 1: H2S reading present in measurement'

/COpresent 'Tag 1: CO reading present in measurement'

/LELpresent 'Tag 1: LEL reading present in measurement'

/O2deviation 'Tag 1: O2 reading different form 20.9 present in measurement'

/R_censored 'Tag 1: Right censored data present in measurement'.

VARIABLE WIDTH H2S_negative(6) H2S_positive(6) H2S_hi(6) interval0 (6) Interval2 (6)

/median_dur (6) SD_interval2 (6) Calc_dur(7) H2Spresent(6) COpresent(6) LELpresent(6) O2deviation(6)

/R_censored(6) Dur_check(8) FullData(6) FullDay(6).

FORMATS H2S_negative(f1.0) H2S_positive(f1.0) H2S_hi(F1.0) Interval0(F4.2) Fulldata(F1.0)

/FullDay(F1.0) H2S_positive(f1.0) H2Spresent(f1.0) COpresent(f1.0) LELpresent(f1.0) O2deviation(f1.0)

/R_censored(f1.0).

EXECUTE.

*End of part G.

*Part H: Algorithm preparation data: Numbers correspond with original index publication (Austigard 2021).

* 4 ** Identifies exposed time for each LOGID *********.

****** Calculates MA=Centralised Moving Average *******************************.

******* NB! Different syntax for different log intervals *******************************.

DATASET ACTIVATE DataSet1.

USE ALL.

*4.b. Cleans up desimals:.

IF ((interval2>0.23) and (interval2<0.27)) Interval2 =0.25.

EXECUTE.

COMPUTE valid_15sec=0.

EXECUTE.

IF (Interval2 = 0.25) valid_15sec = 1.

VARIABLE LABELS valid_15sec 'Intermediate - Measurement interval 15 sec. 1=yes'.

VARIABLE WIDTH valid_15sec(6).

FORMATS valid_15sec(f1.0).

EXECUTE.

*4.c: Moving avereage - task.

SORT CASES BY LogID1 Run Lnr.

*Originally by Logid1. By putting "Run" instead, multiple runs do not disturb.

SPLIT FILE SEPARATE BY Run.

*/*MA 5+1+6 datapoint = 3 min. If 12 points are not available, down to 3 points will be used,

*leaving only startpoint and endpoint without data.

* misses task if only 1st or last row contains H2S value.

*Leavs a worning message on sorting. Is OK.

CREATE H2S_MA_15sec = MA(new_h2s 12 2).

EXECUTE.

Split file off.

Execute.

*Correction for first and last row missing when H2S data present:.

If (missing(H2S_MA_15sec) and readings=1 and H2S_positive =1) H2S_MA_15sec = new_h2s/3.

EXECUTE.

COMPUTE H2S_MA_3min = H2S_MA_15sec.

EXECUTE.

*Corrections for irregular intervals prior to measured level Short format transfer (Interval2>0,25)

*only apply to zero data.

IF ((Interval2 >1.5) and H2S_Positive =0) H2S_MA_3min =0.

EXECUTE.

VARIABLE LABELS H2S_MA_15sec '3 min moving average if 15 sec rate'

/H2S_MA_3min 'Moving average 3 min'.

VARIABLE WIDTH H2S_MA_15sec (8) H2S_MA_3min (8).

** 5.0 ** Identifies peak. Differences according to logging interval.

DATASET ACTIVATE DataSet1.

SORT CASES BY LOGID1 Lnr.

SPLIT FILE SEPARATE BY LOGID1.

CREATE

/H2S_lag1=LAG(new_H2S 1)

/H2S_lead1=LEAD(new_H2S 1)

/H2S_MA_5=MA(new_H2S 4)

/H2S_MA_3=MA(new_H2S 2).

EXECUTE.

IF (H2S_Positive =0 & Interval2 >1.5) H2S_MA_5 =0.

IF (H2S_Positive =0 & Interval2 >1.5) H2S_MA_3 =0.

EXECUTE.

If ((missing(H2S_MA_3) and H2S_positive =1)) H2S_MA_3 = new_h2s/3.

EXECUTE.

*effective in interval2 0.5-1.5:. positive values have regular intervals 0.25 min.

IF (missing(H2S_MA_5) or (Interval2>0.5)) H2S_MA_5 = H2S_MA_3*(3/5).

IF ((Interval2>0.5) and (H2S_MA_3min=0)) H2S_MA_3min = H2S_MA_5*(5/12).

*value for 1.25 min adjusted to 3 min mean.

EXECUTE.

COMPUTE Peak01 = 0.

COMPUTE Fraction = 0.

IF (H2S_MA_5>0) Fraction = new_H2S / H2S_MA_5.

IF (Missing(Interval2) and Fraction >0) Peak01=1.

Execute.

IF (new_H2S > H2S_lag1 and new_H2S >H2S_lead1) Peak01 =1.

IF (Missing(H2S_lag1) and (new_H2S > H2S_lead1)) Peak01 =1.

IF (Missing(H2S_lead1) and (new_H2S > H2S_lag1)) Peak01 =1.

/*marks peaks; misses if consecutive identical values are highest. Corrected in 6b).

EXECUTE.

IF (Fraction >= 1.25 and Peak01 >= 1) Peak01 = 2.

/*Fraction 1.25 means at least 25% above CMA. Fraction >=2 means the peak is at least 100% over background (MA 1min+1 datapoint).

EXECUTE.

*Marks single positive datapoints as peak, regardless of height.

IF (H2S_positive =1 AND H2S_lag1 =0 AND H2s_lead1 =0) Peak01 =2.

IF (H2S_positive =1 AND H2S_lag1 =0 AND H2s_lead1 =0) Fraction =5.

EXECUTE.

VARIABLE LABELS H2S_lag1 'Intermediate - H2S Consecutive value'

/H2S_lead1 'Intermediate - H2S Previous value'

/H2S_MA_5 'Intermediate - Moving average for evaluation of peak - 5 points'

/H2S_MA_3 'Intermediate - Moving average for evaluation of peak in redused dataset - 3 points'

/Peak01 'Peak: 0=no 1=higher than both neighbours 2=yes'

/Fraction 'Relative level to CMA'.

VARIABLE WIDTH H2S_lag1 (8) H2S_lead1(8) H2S_MA_5 (8) H2S_MA_3(8)

/Peak01(4) Fraction(8).

FORMATS Peak01 (f1.0).

SPLIT FILE OFF.

EXECUTE.

** 6 ** Identifies and count insidences of positiv MA (=task) .

DATASET ACTIVATE DataSet1.

USE ALL.

SORT CASES BY LOGID1 Lnr(A).

SPLIT FILE SEPARATE BY LOGID1.

COMPUTE exposed_task = 0.

IF (H2S_MA_3min > 0) exposed_task = 1.

IF (H2S_positive =1 AND H2S_lag1 =0 AND H2s_lead1 =0) exposed_task =1.

FORMATS exposed_task (f1.0).

EXECUTE.

COMPUTE task = 0. /*Only the first datapoint in each task is marked.*Fails if task starts i first log.

CREATE

/task=DIFF(exposed_task 1).

IF (task= -1) task = 0.

EXECUTE.

*Correction for first log:.

If (missing(task) and (exposed_task =1)) Task=1.

EXECUTE.

COMPUTE task_id =0. /*Each datapoint in the same task is marked with the same number.

CREATE

/task_id=CSUM(task).

IF (exposed_task = 0) task_id = 0.

EXECUTE.

SPLIT FILE OFF.

EXECUTE.

** 6a * Makes an unique job ID: task_id1. no decimals means unexposed data.

*Manage up to 1000 tasks, inkl a 0 task ***.

COMPUTE task_id1 = 0.

COMPUTE task_id1 = LOGID1+task_id/1000.

EXECUTE.

VARIABLE LABELS exposed_task 'Exposed task: 0=no, 1=yes'

/task 'Marks start point of tasks for counting'

/task_id 'Tasknumber within measurement'

/task_id1 'Identifikation number measurement and task before correcting irregular logs'.

FORMATS Task_ID1(f5.3).

** 6b ** Finds MAX H2S for each task. Adds it to the dataset ******************.

DATASET ACTIVATE DataSet1.

USE ALL.

SORT CASES BY Task_ID1 Lnr.

*Inserts and updates level each time the data gives a higher H2S-level within a task-id2.

AGGREGATE

/OUTFILE=* MODE=ADDVARIABLES OVERWRITEVARS=YES

/PRESORTED

/BREAK=task_id1

/h2s_task_max=MAX(new_h2s)

/task_countpos=SUM(H2S_positive).

EXECUTE.

*/ securing marker for 1 peak in task also if there are multiple consecutive values on peak level.

IF (not(H2S_lead1=new_H2S) AND (H2S_task_max = new_H2S) and (task_id>0)) Peak01=2.

IF (not(H2S_lead1=new_H2S) AND (H2S_task_max = new_H2S) and (task_id>0)) Fraction=5.

EXECUTE.

*/Removing "tasks" that are due to irregular intervals:.

IF Task_countpos =0 Task =0.

IF Task_countpos =0 Exposed_task =0.

IF H2s_task_max =0 task =0.

EXECUTE.

DELETE VARIABLES Task_Id h2s_task_max.

SORT CASES BY LOGID1 Lnr(A).

SPLIT FILE SEPARATE BY LOGID1.

COMPUTE task_id =0. /*Ready for updating.

CREATE

/task_id=CSUM(task).

IF (exposed_task = 0) task_id = 0.

EXECUTE.

IF (task_ID=0) H2S_MA_3min =0.

EXECUTE.

COMPUTE task_id2 = 0.

COMPUTE task_id2 = LOGID1+task_id/1000.

EXECUTE.

VARIABLE LABELS task_id2 'Identifikation number measurement and task'.

formats Task_ID2(F5.3).

EXECUTE.

SORT CASES BY task_id2 Lnr.

AGGREGATE

/OUTFILE=* MODE=ADDVARIABLES OVERWRITEVARS=YES

/PRESORTED

/BREAK=task_id2

/h2s_task_max=MAX(new_h2s) /*Inserts and updates level each time the data gives a higher H2S-level within a task-id2.

/Time_taskID2=sum(Interval2)

/N_Task=N. /*Count the number of datapoints in each measurement in each task_ID2.

EXECUTE.

If (missing(H2S_task_max)) H2S_task_max =0.

EXECUTE.

*Time_exposed_task, H2S_taskmax2 and total_task_time, only recorded on startpoint of task:.

DO IF task=1.

+ Compute Time_exposed_task = 0.

+ Compute Time_exposed_task = Time_taskID2.

+ compute H2S_taskmax2 = 0.

+ compute H2S_taskmax2 = H2S_task_max.

END IF.

Execute.

*Correcting task-length to minimum 3 min.

DO IF(task=1 and N_task<12).

+ COMPUTE Time_exposed_task=((11+n_task)*0.25).

END IF.

EXECUTE.

*Correcting task length to minimum 3 minutes.

DO IF (Task=1 and Time_exposed_task <3).

+ compute Time_exposed_task =3.

End if.

EXECUTE.

Compute Total_task_time0 = 0.

Execute.

AGGREGATE

/OUTFILE=* MODE=ADDVARIABLES OVERWRITE = yes

/BREAK=LOGID1

/Total_task_time0 =sum(Time_exposed_task).

EXECUTE.

DO IF diff=1.

+ Compute total_tasktime_logid = Total_task_time0.

END IF.

EXECUTE.

*DELETE VARIABLES Total_task_time0.

FORMATS Time_taskID2(F5.2) h2s_task_max(f3.1) N_task(f8.0).

VARIABLE LABELS Time_taskID2 'time in minutes'

/h2s_taskmax2 'Maximum H2S value in task'

/N_task 'Number of datapoints in task'

/Time_taskid2 'Duration of the task in min'

/Time_exposed_task 'Duration of task in min - only recorded once in each task'

/Total_tasktime_LogID 'Total duration of task per LogID in min - recorded once in each LogID'.

EXECUTE.

*End of part H.

*Part I: Algorithm generation:.

** 7 ** Finds level of exposure in intervals according to published index ****.

DATASET ACTIVATE DataSet1.

SORT CASES BY LOGID1 task_id2.

SPLIT FILE SEPARATE BY LOGID1.

COMPUTE n_h2s01 = 0.

COMPUTE n_h2s1 = 0.

COMPUTE n_h2s5 = 0.

COMPUTE n_h2s10 =0.

COMPUTE n1_h2s01 = 0.

COMPUTE n1_h2s5 = 0.

FORMATS n_h2s01(f1.0) n_h2s1(f1.0) n_h2s5(f1.0) n_h2s10(f1.0) n1_h2s01(f1.0) n1_h2s5(f1.0).

VARIABLE LABELS n_h2s01 'Intermediate: new_H2S is within interval 0 - 1.0 0=no 1=yes'

/n_h2s1 'Intermediate: new_H2S is within interval 1.1-5.0 0=no 1=yes'

/n_h2s5 'Intermediate: new_H2S is within interval 5.1 - 10.0 0=no 1=yes'

/n_h2s10 'Intermediate: new_H2S is within interval >10 0=no 1=yes'

/n1_h2s01 'Intermediate:Datapoint in LOGID that is =<5.0 ppm. 0=no 1=yes'

/n1_h2s5 'Intermediate: Datapoint in LOGID that is >5.0 ppm. 0=no 1=yes'.

EXECUTE.

** 7a * Gives value 1 if new_H2S is in the current interval, else 0 ****.

*Desimals according to equipment. Ours give 1 decimal, so we use 2.

COUNT n_H2S01=new_h2s(0.001 thru 1.00).

COUNT n_H2S1=new_h2s(1.01 thru 5.00).

COUNT n_H2S5=new_h2s(5.01 thru 10.00).

COUNT n_H2S10=new_h2s(10.01 thru 1000).

COUNT n1_H2S01=new_H2S(0.001 thru 5.00).

COUNT n1_H2S5=new_H2S(5.01 thru 1000).

EXECUTE.

** 7b * Gives value 1 if peak in current interval, else 0 ****.

COMPUTE h2s01_count0 =0.

COMPUTE h2s1_count0 =0.

COMPUTE h2s5_count0 =0.

COMPUTE h2s10_count0 =0.

FORMATS h2s01_count0(f1.0) h2s1_count0(f1.0) h2s5_count0(f1.0) h2s10_count0(f1.0).

VARIABLE LABELS h2s01_count0 'Tag of peak in interval 0-1.0. 0=no 1=yes'

/h2s1_count0 'Tag of peak in interval 1.1-5.0ppm. 0=no 1=yes'

/h2s5_count0 'Tag of peak in interval 5.1-10.0. 0=no 1=yes'

/h2s10_count0 'Tag of peak in interval >10ppm. 0=no 1=yes'.

IF (n_H2S01=1 & Peak01=2) h2s01_count0=1.

IF (n_H2S1=1 & Peak01=2) h2s1_count0=1.

IF (n_H2S5=1 & Peak01=2) h2s5_count0=1.

IF (n_H2S10=1 & Peak01=2) h2s10_count0=1.

EXECUTE.

** 7c * Finds number of counts for each element of the index for each LOGID ***.

SPLIT FILE off.

COMPUTE h2s01_count =0.

COMPUTE h2s1_count =0.

COMPUTE h2s5_count =0.

COMPUTE h2s10_count =0.

FORMATS h2s01_count(f1.0) h2s1_count(f1.0) h2s5_count(f1.0) h2s10_count(f1.0).

VARIABLE LABELS h2s01_count 'Number of peaks in interval per LogID 0-1.0'

/h2s1_count 'Number of peaks in interval per LogID 1.1-5.0ppm'

/h2s5_count 'Number of peaks in interval per LogID 5.1-10.0'

/h2s10_count 'Number of peaks in interval per LogID >10ppm'.

EXECUTE.

DO IF (Logid1 >0 and H2Spresent =1).

+COMPUTE datapoint_M_mean= 0.

+Compute Datapoint_M_Mean = new_h2s*interval2/(Time_logid_full*60).

End if.

EXECUTE.

*Time_LogID might be to long to represent true runtime. Uses LogRunTime0 instead. It is on every line, oposite

*to LogRunTime wich is only on 1. datapoint of LogID.

AGGREGATE

/OUTFILE=* MODE=ADDVARIABLES OVERWRITE = yes

/BREAK=LOGID1

/h2s01_count =sum(h2s01_count0)

/h2s1_count = sum(h2s1_count0)

/h2s5_count = sum(h2s5_count0)

/h2s10_count = sum(h2s10_count0)

/h2s_logid_max = max(new_h2s)

/h2s_logid_mean = sum(datapoint_M_mean)

/h2s01_count1 =sum(n1_h2s01)

/h2s5_count1 = sum(n1_h2s5).

FORMATS H2S_logid_max (F4.2) H2S_logid_mean (F4.2) h2s01_count1(f2.0) h2s5_count1(f2.0).

VARIABLE LABELS datapoint_M_mean 'Weighted datapoint attribution to mean in measurement'

/h2s01_count 'Number of datapoints per LOGID in interval<= 1.0 ppm'

/h2s1_count 'Number of datapoints per LOGID in interval1.1 -5.0 ppm'

/h2s5_count 'Number of datapoints per LOGID in interval 5.1 - 10.0 ppm'

/h2s10_count 'Number of datapoints per LOGID in interval>10.0 ppm'

/h2s_logid_max 'Maximum H2S-level in LOGID'

/h2s_logid_mean 'Mean H2S-level in LOGID'

/h2s01_count1 'Number of measurement points per LOGID in interval <=5.0 ppm'

/h2s5_count1 'Number of measurement points per LOGID in interval >5.0 ppm'.

If missing(H2S_logid_max) H2S_logid_max=0.

Execute.

DATASET ACTIVATE DataSet1.

Filter OFF.

Use ALL.

COMPUTE Time_positive = 0.

*Modification for not equal interval througout measurement. Readings of value different from 0 have

/ duration 0.25.If only full data, 0.25 replace by "Interval2".

COMPUTE Time_pos01 = h2s01_count1*0.25.

COMPUTE Time_pos5 = h2s5_count1*0.25.

EXECUTE.

COMPUTE Time_positive =Time_pos01 + Time_pos5.

COMPUTE Positive_counts =h2s01_count1+h2s5_count1.

EXECUTE.

VARIABLE LABELS Time_pos01 'Duration in min <= 5 ppm'

/Time_pos5 'Duration in min > 5 ppm'

/Time_positive 'Total positive time in min per LogID'

/Positive_counts 'Total number of positive counts per LogID'.

EXECUTE.

** 8 ** Calculates H2S_index ************************************************.

DATASET ACTIVATE DataSet1.

SORT CASES BY LOGID1 Lnr.

SPLIT FILE SEPARATE BY LOGID1.

COMPUTE n_h2s_index = 0.

COMPUTE n_h2s_index_1 = h2s01_count*0.1.

COMPUTE n_h2s_index_2 = h2s1_count.

COMPUTE n_h2s_index_3 = h2s5_count*5.

COMPUTE n_h2s_index_4 = h2s10_count*10.

COMPUTE n_h2s_index_5 = h2s_logid_max.

COMPUTE n_h2s_index_6 = Time_pos01*0.1.

COMPUTE n_h2s_index_7 = Time_pos5*5.

COMPUTE n_h2s_index =n_h2s_index_1 + n_h2s_index_2 + n_h2s_index_3 + n_h2s_index_4 + n_h2s_index_5 + n_h2s_index_6 + n_h2s_index_7.

IF LOGID1=0 n_h2s_index =0.

VARIABLE LABELS n_h2s_index 'Index value for measurement'

/n_h2s_index_1 'Index element: (number of peaks=<1 ppm)*0.1'

/n_h2s_index_2 'Index element: (number of peaks 1.1 - 5.0 ppm)*1'

/n_h2s_index_3 'Index element: (number of peaks 5.1 - 10.0 ppm)*5'

/n_h2s_index_4 'Index element: (number of peaks >10 ppm)*10'

/n_h2s_index_5 'Index element: maximum H2S level in measurement'

/n_h2s_index_6 'Index element: (Time =< 5 ppm)*0.1'

/n_h2s_index_7 'Index element: (Time > 5 ppm)*5'.

SPLIT FILE OFF.

FILTER OFF.

USE all.

EXECUTE.

** End part I: of syntax to convert measurement data to index value *****************

*Part J: Other calculations:

*9.a. TWA.

*Compute TWA_8 = (h2s_logid_mean* N_logid * Interval2)/480.

*If most 0 datapoints are left out:.

COMPUTE TWA_8 = (h2s_logid_mean * time_LogID_full*60)/480.

Compute TWA_hi =0.

IF TWA_8>1 TWA_hi =1.

EXECUTE.

VARIABLE LABELS TWA_8 '8 hour time weighted average for measurment'

/TWA_hi 'TWA >20% of OEL'.

EXECUTE.

*9.b. Part of measurement time under level of detection.

Do if (LogID1>0 and Diff=1).

+COMPUTE LOD_timeAlgorithm = 0.

+COMPUTE LOD_timeAlgorithm = time_logID - Time_pos01 - Time_pos5.

+COMPUTE LOD_A_percent =0.

+COMPUTE LOD_A_percent = LOD_timeAlgorithm *100/ Time_logID.

End if.

EXECUTE.

FORMATS LOD_timeAlgorithm(f3.1).

VARIABLE LABELS LOD_timeAlgorithm 'Time in LogID below LOD'

/LOD_A_percent 'Percetage of time in measurement below LOD'.

EXECUTE.

*9.c. Time from start to peak and time from peak to shutdown. Evaluating late turn-on and early turn-of.

*tagged only at detected peaks if criteria are met. Indication of more possible exposure.

EXECUTE.

SORT CASES by logID1 Lnr.

EXECUTE.

DO IF Peak01 =2.

+Compute TimeToPeak=0.

+Compute TimeToPeak = rnd(time - Runtime_min)/60.

+Compute TimeToShutdown=0.

+Compute TimeToShutDown = rnd(Runtime_max - time)/60.

END IF.

Execute.

DO IF (Peak01=2 and TimeToPeak<3 ).

+Compute LateTurnOn3 =1.

END IF.

EXECUTE.

DO IF (Peak01=2 and TimeToPeak<1 ).

+Compute LateTurnOn1 =1.

END IF.

EXECUTE.

DO IF (Peak01=2 and TimeToShutDown <3).

+COMPUTE EarlyTurnOff3= 1.

END IF.

EXECUTE.

DO IF (Peak01=2 and TimeToShutDown <1).

+COMPUTE EarlyTurnOff1= 1.

END IF.

EXECUTE.

VARIABLE LABELS TimeToPeak 'Time from run-start to peak'

/TimeToShutDown 'Time from peak to run-end'

/LateTurnOn3 'Turned on <3 min prior to exposure'

/LateTurnOn1 'Turned on <1 min prior to exposure'

/EarlyTurnOff3 'Turned off <3 min after exposure'

/EarlyTurnOff1 'Turned off <1 min after exposure'.

FORMATS TimeToPeak(F4.0) TimeToShutDown(F4.0) LateTurnOn3(F1.0) LateTurnOn1(F1.0)

/EarlyTurnOff3(F1.0) EarlyTurnOff1(F1.0).

FILTER OFF.

SPLIT FILE OFF.

*End of part J.

*Part K. Aggregating LogID2-data.

*GET FILE='Filepath/Filename.sav'.

** 10a making a file aggregated on ID.

*Aggergated level (not giving long measurements extra weight).

DATASET ACTIVATE DataSet1.

FILTER off.

USE ALL.

COMPUTE LOGID_not_0 =1.

IF LOGID1 = 0 LOGID_not_0 =0.

FILTER by LOGID_not_0.

SORT CASES BY ID LOGID1 Lnr.

*When aggregating data: .

*MIN : when minimum data is recorded.

*MEAN : Mean value, but also used for parameters with same value in every row

*MAX : when maximum data is recorded, for example data only once for every LogId1.

*MEDIAN: To compare with MEAN when differences excists.

DATASET DECLARE AggrIndex0.

AGGREGATE

/OUTFILE='AggrIndex0'

/PRESORTED

/BREAK=LOGID1

/ID = MEAN(ID)

/Year =MEAN(Year)

/Month = MEAN(Month)

/Day = MEAN(Day)

/Weekday = max(weekday)

/DayOfYear =mean(Time_day)

/starttime =MIN(time)

/endtime =MAX(Time)

/CorrTime_min =min(CorrTime_min)

/CorrTime_max = max(CorrTime_max)

/N_LnrID_sum = max(N_LnrID_sum)

/N_logid0 =max(N_logid0)

/N_logid = Max(N_logid)

/Run_inLogID = max(Run_inlogID)

/RunN_min = min(RunN)

/RunN_mean = Mean(RunN)

/RunN_max = Max(runN)

/LongRun = max(LongRun)

/Runreading_min =min(RunreadingN)

/Runreading_mean = mean(RunreadingN)

/Runreading_max = max(RunreadingN)

/h2s_min=MIN(new_h2s)

/h2s_mean=MEAN(h2s_logid_mean)

/h2s_median = MEDIAN(new_h2s)

/h2s_logid_max = MAX(h2s_logid_max)

/TWA_8 = MEAN(TWA_8)

/TWA_hi = max(TWA_hi)

/h2s01_count = MEAN(h2s01_count)

/h2s1_count = MEAN(h2s1_count)

/h2s5_count = MEAN(h2s5_count)

/h2s10_count = MEAN(h2s10_count)

/h2s01_count1 = MEAN(h2s01_count1)

/h2s5_count1 =MEAN(h2s5_count1)

/n_h2s_index=MEAN(n_h2s_index)

/n_h2s_index_1=MEAN(n_h2s_index_1)

/n_h2s_index_2=MEAN(n_h2s_index_2)

/n_h2s_index_3=MEAN(n_h2s_index_3)

/n_h2s_index_4=MEAN(n_h2s_index_4)

/n_h2s_index_5=MEAN(n_h2s_index_5)

/n_h2s_index_6=MEAN(n_h2s_index_6)

/n_h2s_index_7=MEAN(n_h2s_index_7)

/time_LOGID = MEAN(time_LOGID)

/Calc_dur = max(Calc_dur)

/dur_check = max(dur_check)

/median_interval = max(median_dur)

/mean_interval= mean(interval2)

/SD_interval = mean(SD_interval2)

/N_positive = sum(H2S_positive)

/PosData = max(H2S_positive)

/R_censored = max(R_censored)

/FullData = max(FullData)

/FullDay = max(FullDay)

/LOD_timeAlgorithm = mean(LOD_timeAlgorithm)

/LOD_A_percent = mean(LOD_A_percent)

/Time_positive = max(Time_positive)

/RunInLog =sum(rundiff)

/No_of_tasks = sum(task)

/Positive_counts = max(positive_counts)

/tasktime_min =min(Time_exposed_task)

/Tasktime_mean = mean (Time_exposed_task)

/Tasktime_median = median(Time_exposed_task)

/tasktime_max =max(Time_exposed_task)

/total_tasktime_LogID = max(Total_tasktime_logid)

/H2S_task_max_min = min(H2S_taskmax2)

/H2S_task_max_mean = mean(H2S_taskmax2)

/H2S_task_max_median = median(H2S_taskmax2)

/H2S_task_max_max = max(H2S_taskmax2)

/PowerUp = sum(powerUp)

/ShutDown = sum(ShutDown)

/H2Spresent = max(H2Spresent)

/COpresent =max(COpresent)

/LELpresent =max(LELpresent)

/O2deviation =max(O2deviation)

/Sensors_data =max(sensors_data)

/BumpPresent = max(BumpPresent)

/CalibrationPresent = max(calibrationPresent)

/LastH2SCal = max(LastH2SCal)

/LastH2SBump = max(LastH2SBump)

/DaysfromBump = min(DaysFromBump)

/DaysfromCal =min(DaysFromCal)

/bumpCalGenerated = max(BumpCalGenerated)

/min_TimeToPeak = min(timeToPeak)

/min_TimeToShutDown =min(timeToShutDown)

/LateTurnOn3 = max(LateTurnOn3)

/LateTurnOn1 = max(LateTurnOn1)

/LateTurnOn_sum = sum(lateTurnOn3)

/EarlyTurnOff3 = Max(earlyTurnOff3)

/EarlyTurnOff1 =Max(EarlyTurnOff1)

/EarlyTurnOff_sum =Sum(EarlyTurnOff3)

/Diff = max(diff).

EXECUTE.

DATASET ACTIVATE AggrIndex0 WINDOW=FRONT.

DATASET NAME AggrIndex0 WINDOW=FRONT.

VARIABLE WIDTH Year(6) Month(6) Day(6).

FORMATS ID(F4.0) Year(F4.0) Month(F2.0) Day(F2.0) PowerUp(F1.0) ShutDown (F1.0) Weekday(F1.0) TWA_8(F7.4).

FORMATS h2s01_count(F3.0) h2s1_count(F3.0) h2s5_count(F3.0) h2s10_count(F3.0).

FORMATS h2s01_count1(F3.0) h2s5_count1(F3.0) RunInLog(F3.0) Positive_counts(F3.0).

* More than 2 decimals in TWA_8 must be set manually.

VARIABLE LABELS ID 'Person identifier'

/Year 'Year'

/Month 'Month'

/Day 'Day'

/Weekday 'Weekday, starting with 1=sunday'

/N_LnrID_sum 'Total nr of rows in ID'

/RunN_min 'Minimum nr of logs in run in meassurement'

/RunN_Mean 'Mean nr of logs in run in meassurement'

/RunN_max 'Maximum nr of logs in run in meassurement'

/Runreading_min 'Min nr of readings in runs in LogID'

/Runreading_mean 'Mean nr of readings in runs in LogID'

/Runreading_max 'Max nr of readings in runs in LogID'

/LongRun 'LogID part of av LongRun'

/h2s_min 'Min of measured level'

/h2s_mean 'Mean of measurement (weighted)'

/h2s_median 'Median of measurement (not weighted)'

/TWA_8 'TWA 8 hour average'

/h2s01_count 'Number of peaks per LOGID in interval<= 1.0 ppm'

/h2s1_count 'Number of peaks per LOGID in interval1.1 -5.0 ppm'

/h2s5_count 'Number of peaks per LOGID in interval 5.1 - 10.0 ppm'

/h2s10_count 'Number of peaks per LOGID in interval>10.0 ppm'

/h2s01_count1 'Number of measurement points per LOGID in interval <=5.0 ppm'

/h2s5_count1 'Number of measurement points per LOGID in interval >5.0 ppm'

/n_h2s_index 'Index value for measurement'

/n_h2s_index_1 'Index element: number of peaks=<1 ppm'

/n_h2s_index_2 'Index element: number of peaks 1.1 - 5.0 ppm'

/n_h2s_index_3 'Index element: number of peaks 5.1 - 10.0 ppm'

/n_h2s_index_4 'Index element: number of peaks >10 ppm'

/n_h2s_index_5 'Index element: maximum H2S level in measurement'

/n_h2s_index_6 'Index element: Time =< 5 ppm'

/n_h2s_index_7 'Index element: Time > 5 ppm'

/time_LOGID 'Duration (minutes)'

/Calc_dur 'Control calculation of duration'

/median_interval 'Median of log interval'

/mean_interval 'Mean of log interval'

/sd_interval 'SD of log interval'

/N_positive 'Nr of positive logs in measurement'

/PosData '1 = positive data in measurement'

/LOD_timeAlgorithm 'Time in measurement below LOD'

/LOD_A_percent 'Percent time below LOD'

/RunInLog 'Nr of runs in LogID2'

/No_of_tasks 'Nr of tasks in measurement'

/tasktime_min 'Task time minimum (exposed)'

/tasktime_mean 'Task time mean (exposed)'

/tasktime_median 'Task time median (exposed)'

/tasktime_max 'Task time max (exposed)'

/total_tasktime_LogID 'Sum of task time in LogID'

/H2S_task_max_min 'Lowest task max in LogID'

/H2S_task_max_mean 'Mean task max in LogID'

/H2S_task_max_median 'Median task max in LogID'

/H2S_task_max_max 'Highest task max in LogID'

/PowerUp 'Sum of PowerUp tag in data'

/ShutDown 'Sum of ShutDown tag in data'

/Min_TimeToPeak 'Min Time from run-start to peak'

/Min_TimeToShutDown 'Min time from peak to ShutDown'

/LateTurnOn_sum 'one or more late turnOns in LogID'

/EarlyTurnOff_sum 'One or more early turnOffs in LogID'

/Diff 'count for new measurement'.

COMPUTE LogID = 0.

COMPUTE LogID2 =0.

COMPUTE LogID2 = $casenum.

Compute LOD_level =1.6.

EXECUTE.

FORMATS LogID (f6.0) LogID2(F4.0) LOD_level(F3.1).

VARIABLE LABELS LogID 'Consecutive total measurements, all IDs'

/LogID2 'Consecutive total measurements, person'

/LOD_level 'LOD-level of detection (ppm)'.

*Store individual graps for follow up?.

* Also: Amount of data in Output gets to large after some runs - it slows down the algorithm. clear it occationaly.

OUTPUT CLOSE *.

List

/Variables ID

/CASES from 1 to 1.

SORT CASES by Year month day ID.

Split file by Year.

CREATE

/LogIDYear=CSUM(Diff).

EXECUTE.

Split file off.

EXECUTE.

FORMATS LogIDYear(F3.0) .

VARIABLE LABELS LogIDYear 'Consecutive number for person in a year'.

* LogLinear x- and y-axis.:.

GGRAPH

/GRAPHDATASET NAME="graphdataset" VARIABLES=TWA_8 n_h2s_index MISSING=LISTWISE

REPORTMISSING=NO

/GRAPHSPEC SOURCE=INLINE

/FITLINE TOTAL=YES.

BEGIN GPL

SOURCE: s=userSource(id("graphdataset"))

DATA: n_h2s_index=col(source(s), name("n_h2s_index"))

DATA: TWA_8=col(source(s), name("TWA_8"))

GUIDE: axis(dim(1), label("H2S index from algorithm"))

GUIDE: axis(dim(2), label("TWA 8 hour (ppm)"))

GUIDE: text.title(label("Simple Scatter of TWA 8 hour average by Index value for measurement"))

SCALE: log(dim(2), min(0.0001), max(6))

SCALE: log(dim(1), base(10), max(1000))

ELEMENT: point(position(n_h2s_index*TWA_8))

END GPL.

*Log lineær regresjon:

* 11a TWA to Index-graph - linear x- and y-xis.

GGRAPH

/GRAPHDATASET NAME="graphdataset" VARIABLES=TWA_8 n_h2s_index MISSING=LISTWISE

REPORTMISSING=NO

/GRAPHSPEC SOURCE=INLINE

/FITLINE TOTAL=YES.

BEGIN GPL

SOURCE: s=userSource(id("graphdataset"))

DATA: n_h2s_index=col(source(s), name("n_h2s_index"))

DATA: TWA_8=col(source(s), name("TWA_8"))

GUIDE: axis(dim(1), label("H2S index from algorithm"))

GUIDE: axis(dim(2), label("TWA 8 hour (ppm)"))

ELEMENT: point(position(n_h2s_index*TWA_8))

END GPL.

/* linear regression.

UNIANOVA

TWA_8 WITH n_h2s_index

/METHOD = SSTYPE(3)

/INTERCEPT = INCLUDE

/PRINT = PARAMETER

/CRITERIA = ALPHA(.05)

/DESIGN = n_h2s_index.

EXECUTE.

FREQUENCIES BumpCalGenerated lastH2SCal LastH2SBump.

DESCRIPTIVES VARIABLES= N_logid0 N_logid time_LOGID No_of_tasks total_tasktime_logid H2S_task_max_max

/STATISTICS=MEAN SUM MIN MAX.

*Full graph H2S.

DATASET ACTIVATE Dataset1.

EXECUTE.

* Chart Builder.

GGRAPH

/GRAPHDATASET NAME="graphdataset" VARIABLES=LogTime new_h2s MISSING=LISTWISE REPORTMISSING=NO

/GRAPHSPEC SOURCE=INLINE.

BEGIN GPL

SOURCE: s=userSource(id("graphdataset"))

DATA: LogTime=col(source(s), name("LogTime"))

DATA: new_h2s=col(source(s), name("new_h2s"))

GUIDE: axis(dim(1), label("Log Time"))

GUIDE: axis(dim(2), label("Measured level of H2S"))

GUIDE: text.title(label("Overwiev of measured levels. Full measurement periode"))

ELEMENT: line(position(LogTime*new_h2s), missing.wings())

END GPL.

*Store parts of output for individual follow up:.

OUTPUT EXPORT

/CONTENTS EXPORT = VISIBLE

/DOC

DOCUMENTFILE = 'Filepath\Profil_ID.doc'

PAGESIZE =MM(210, 297)

TOPMARGIN = MM(10)

BOTTOMMARGIN = MM(10)

LEFTMARGIN =MM(10)

RIGHTMARGIN =MM(10).

*****2nd run and on: Merging aggregated files.

DATASET ACTIVATE Aggr_Index_all WINDOW=FRONT.

ADD FILES /FILE=*

/FILE=AggrIndex0.

EXECUTE.

COMPUTE LogID = $casenum.

EXECUTE.

*SAVE OUTFILE='Filepath\Aggr_Index_all02.sav'

/COMPRESSED.

DATASET CLOSE AggrIndex0.

* save positive H2S readings in dedicated file.

DATASET ACTIVATE DataSet1.

DATASET COPY Positive_Readings0.

DATASET ACTIVATE Positive_Readings0.

FILTER OFF.

USE ALL.

SELECT IF (H2S_positive =1).

EXECUTE.

DATASET ACTIVATE Positive_Readings.

ADD FILES /FILE=*

/FILE='Positive_Readings0'.

EXECUTE.

*SAVE OUTFILE='Filepath\Positive_Readings02.sav'

/COMPRESSED.

* save positive readings regardless of type in dedicated file.

DATASET ACTIVATE DataSet1.

DATASET COPY Positive_other_Readings0.

DATASET ACTIVATE Positive_other_Readings0.

FILTER OFF.

USE ALL.

SELECT IF (Sensors_data =1).

EXECUTE.

DATASET ACTIVATE Positive_other_Readings.

ADD FILES /FILE=*

/FILE='Positive_other_Readings0'.

EXECUTE.

*SAVE OUTFILE='Filepath\Positive_other_Readings02.sav'

/COMPRESSED.

DATASET CLOSE Positive_Readings0.

DATASET CLOSE Positive_other_Readings0.

DATASET CLOSE Dataset1.

*Run again from line 169 with correkt inputfile and ID till end.
